# Targeting ubiquitin-specific protease 8 sensitizes anti-programmed death-ligand 1 immunotherapy of pancreatic cancer

**DOI:** 10.1038/s41418-022-01102-z

**Published:** 2022-12-20

**Authors:** Hanshen Yang, Xiaozhen Zhang, Mengyi Lao, Kang Sun, Lihong He, Jian Xu, Yi Duan, Yan Chen, Honggang Ying, Muchun Li, Chengxiang Guo, Qingsong Lu, Sicheng Wang, Wei Su, Tingbo Liang, Xueli Bai

**Affiliations:** 1 Department of Hepatobiliary and Pancreatic Surgery, the First Affiliated Hospital, School of Medicine, Zhejiang University, Hangzhou, Zhejiang China; 2 Zhejiang Provincial Key Laboratory of Pancreatic Disease, School of Medicine, Zhejiang University, Hangzhou, Zhejiang China; 3 Innovation Center for the Study of Pancreatic Diseases, Hangzhou, Zhejiang China; 4 Zhejiang Clinical Research Center of Hepatobiliary and Pancreatic Disease, Hangzhou, Zhejiang China; 5 Zhejiang University Cancer Center, Hangzhou, Zhejiang China; 6 Research Center for Healthcare Data Science, Zhejiang Lab, Hangzhou, Zhejiang China

**Keywords:** Oncogenes, Immune evasion

## Abstract

Programmed death-1 (PD-1) and its ligand programmed death-ligand 1 (PD-L1) help tumor cells evade immune surveillance, and are regarded as important targets of anti-tumor immunotherapy. Post-translational modification of PD-L1 has potential value in immunosuppression. Here, we identified that ubiquitin-specific protease 8 (USP8) deubiquitinates PD-L1. Pancreatic cancer tissues exhibited significantly increased USP8 levels compared with those in normal tissues. Clinically, the expression of USP8 showed a significant association with the tumor-node-metastasis stage in multiple patient-derived cohorts of pancreatic cancer. Meanwhile, USP8 deficiency could reduce tumor invasion and migration and tumor size in an immunity-dependent manner, and improve anti-tumor immunogenicity. USP8 inhibitor pretreatment led to reduced tumorigenesis and immunocompetent mice with *Usp8* knockdown tumors exhibited extended survival. Moreover, USP8 interacted positively with PD-L1 and upregulated its expression by inhibiting the ubiquitination-regulated proteasome degradation pathway in pancreatic cancer. Combination therapy with a USP8 inhibitor and anti-PD-L1 effectively suppressed pancreatic tumor growth by activation of cytotoxic T-cells and the anti-tumor immunity was mainly dependent on the PD-L1 pathway and CD8 + T cells. Our findings highlight the importance of targeting USP8, which can sensitize PD-L1-targeted pancreatic cancer to immunotherapy and might represent a novel therapeutic strategy to treat patients with pancreatic tumors in the future.

## Introduction

The most frequently diagnosed form of pancreatic cancer is pancreatic ductal carcinoma (PDAC), which displays high morbidity and mortality, and patients with PDAC have poor prognosis [1]. Recently, advances in immunotherapeutic strategies have raised the expectations for improved treatment outcomes, especially targeted therapy of the important immune checkpoint proteins programmed death protein-1 (PD-1) and its ligand programmed death-ligand 1 (PD-L1) [2, 3]. The strategy of PD-L1/PD-1 pathway inhibition has gained approval in multiple types of cancer, such as breast cancer, melanoma, and non-small cell lung cancer [4, 5]. However, PDAC has a robust immunosuppressive tumor microenvironment, which might be the main reason for reduced response to PD-L1/PD-1 blockade therapy [6]. Therefore, it is vital to uncover the regulatory mechanisms of PD-L1, which might contribute to improving the clinical efficacy of PD-L1-based combinatorial therapy.

Posttranslational modifications (PTMs) of PD-L1 have been proven to play critical roles in modulating cancer cell immunosuppression. Recent studies have revealed that targeting PD-L1 PTMs, including phosphorylation, N-glycosylation, and ubiquitination can regulate PD-L1 to enhance antitumor immune responses, thus providing potential therapeutic options [7–9]. PD-L1 is regulated by the ubiquitin/proteasome pathway through E3 ligases and deubiquitinating enzymes, suggesting that targeting PD-L1 ubiquitination might reduce tumor immunosuppression [10]. A study showed that COP9 signalosome 5 (CSN5) can deconjugate NEDD8 ubiquitin-like modifier (NEDD8) from cullin-NEDD8 to play a negative role in ubiquitin enzyme activity [11]. In our group, we have demonstrated that NIMA-related kinase 2 (NEK2) can phosphorylate PD-L1 at T194 and T210, which suppresses ubiquitination-regulated proteasome-mediated PD-L1 degradation to augment the immune evasion ability [12]. In addition, another study in our group revealed that ubiquitin-specific protease 22 (USP22) can deubiquitinate PD-L1 to stabilize the protein, leading to liver cancer immune resistance [13]. Thus, the above research indicated that PD-L1 ubiquitination modification plays a potentially important role in cancer therapy, and targeting this PTM might yield promising antitumor effects in pancreatic cancer.

Ubiquitin-specific protease 8 (USP8) is a deubiquitinating enzyme (DUB) that can catalyze the release of ubiquitin to protect a protein from degradation [14, 15]. USP8 has a vital function in cell proliferation, the cell cycle, and the surface localization of proteins [16–18]. Notably, accumulating evidence suggests that upregulated or mutated USP8 can lead to cancer progression, metastasis, and poor survival by affecting multiple signaling pathways in different types of tumors, including, but not limited to, lung [19], gastric [20], and breast cancers [21, 22]. Nevertheless, no study on the effect of USP8 in pancreatic cancer has been reported. In particular, the biological role of USP8 in antitumor immunity therapy remains unclear. The present study aimed to explore the regulation of PD-L1 by USP8 and its impact on the treatment of pancreatic cancer.

## Materials and methods

### Antibodies, reagents, and plasmids

This study used the following antibodies and reagents: anti-USP8 (27791-1-AP, Proteintech, Rosemont, IL, USA, 1:1000; ab228572, Abcam, Cambridge, MA, USA, 1:500), anti-PD-L1 (66248-1-Ig, Proteintech, 1:1000; 14-5982-82, Thermo Scientific, Waltham, MA, USA, 1:100; 13684, Cell Signaling Technology, Danvers, MA, USA, 1:100; ab213480, Abcam, 1:1000; ab205921, Abcam, 1:1000), anti-Ubiquitin (3933, Cell Signaling Technology, 1:1000), anti-CD8a (98941, Cell Signaling Technology, 1:200), anti-Granzyme B (44153 S, Cell Signaling Technology, 1:100), MG-132 (S2619, Selleck, Houston, TX, USA), Cycloheximide (S7418, Selleck), human USP8 plasmid (Shanghai ObiO Technology, Shanghai, China), human USP8 lentivirus (HG15979-ACGLN, SinoBiological, Beijing, China), mouse USP8 lentivirus (Shanghai ObiO Technology), mouse Kras plasmid (Shanghai ObiO Technology), mouse P53 plasmid (Shanghai ObiO Technology), mouse PD-L1 CRISPR/Cas9 KO plasmid (sc-425636, Santa Cruz Biotechnology, Santa Cruz, CA, USA), DUB-IN-2 (USP8 inhibitor) (HY-50737A, MedChemExpress, Monmouth Junction, NJ, USA), anti-mouse PD-L1 (B7-H1) (BE0101, Bio X Cell, Lebanon, NH, USA, 200 μg per mouse), recombinant human GST (ab70456, Abcam), human USP8-6*His fusion protein (Ag27104, Proteintech), human PD-L1/CD274-glutathione-S-transferase (GST) fusion protein (Ag27104, Proteintech), anti-cleaved caspase-3 (9661, Cell Signaling Technology, 1:400), anti-Ki67 (12202, Cell Signaling Technology, 1:800), anti-Kras (ab221163, Abcam, 1:1000), anti-P53 (ab26, Abcam, 1:1000), anti-GST (2624, Cell Signaling Technology, 1:1000), Epithelial-Mesenchymal Transition (EMT) Antibody Sampler Kit (9782 T, Cell Signaling Technology, 1:1000), anti-EGFR (ab52894, Abcam, 1:1000), anti-HA (51064-2-AP, Proteintech, 1:5000), anti-α-tubulin (AF0001, Beyotime, 1:2000), HRP goat rabbit IgG (A0208, Beyotime Biotechnology, 1:5000 for WB, 1:100 for IHC), HRP goat anti-mouse IgG (A0216, Beyotime Biotechnology, 1:5000 for WB, 1:100 for IHC), goat anti-rabbit IgG (GTX77061, GeneTex, Southern California, USA, 1:2500), goat anti-mouse IgG (GTX26708, GeneTex, 1:2500), Hoechst 33342 (C1027, Beyotime Biotechnology, 1:100), Anti-rabbit IgG (H + L), F(ab’)2 Fragment (Alexa Fluor® 488 Conjugate) (4412, Cell Signaling Technology, 1:400), Anti-mouse IgG (H + L), F(ab’)2 Fragment (Alexa Fluor® 647 Conjugate) (4410, Cell Signaling Technology, 1:400), Brilliant Violet 605 anti-mouse CD45 (103139, Biolegend, San Diego, California, USA, 1:200), Brilliant Violet 785 anti-mouse CD45 (304048, Biolegend, 1:200), APC anti-mouse CD45 (147707, Biolegend, 1:200), FITC anti-mouse CD3 (100203, Biolegend, 1:200), Brilliant Violet 785 anti-mouse CD3 (100231, Biolegend, 1:200), Brilliant Violet 510 anti-mouse CD3 (100233, Biolegend, 1:200), APC/Cy7 anti-mouse CD4 (100413, Biolegend, 1:200), PE/Cy7 anti-mouse CD8a (100722, Biolegend, 1:200), Brilliant Violet 605 anti-mouse CD8a (100744, Biolegend, 1:200), APC anti-mouse CD152(106309, Biolegend, 1:200), PerCP/Cyanine5.5 anti-human/mouse Granzyme B Recombinant (372211, Biolegend, 1:200), Brilliant Violet 605 anti-mouse IFN-γ (505839, Biolegend, 1:200), APC anti-mouse TNF-α (506307, Biolegend, 1:200), PE anti-mouse Perforin (154306, Biolegend, 1:200), Brilliant Violet 510™ anti-mouse CD326 (118231, Biolegend, 1:200), PE anti-mouse CD326 (118205, Biolegend, 1:200), PerCP/Cyanine5.5 anti-mouse H-2Kd (11661725, Biolegend, 1:200), PE anti-mouse H-2Kd (116607, Biolegend, 1:200), PE/Cy7 anti-mouse CD274 (124313, Biolegend, 1:200), PE anti-mouse CD274 (124307, Biolegend, 1:200), APC anti-mouse CD274 (124311, Biolegend, 1:200), APC anti-human CD274 (329707, Biolegend, 1:200), Brilliant Violet 421 anti-human CD274 (329714, Biolegend, 1:200), Trustain FcX anti-mouse CD16/CD32 (101320, Biolegend, 1:200), human TruStain FcX (422302, Biolegend, 1:200), LIVE/DEAD™ Fixable Violet dead cell stain kit with 405 nm excitation (L34955, Thermo Fisher Scientific), Percoll (17-0891-01, GE Healthcare, Connecticut, USA), leukocyte activation cocktail (550583, BD Biosciences, Franklin Lakes, New Jersey, USA), Fixation/Permeabilization solution kit (555028, BD Biosciences), calcium chloride solution (21115, Sigma-Aldrich, St. Louis, MO, USA), collagenase IV (17104019, Thermo Fisher Scientific), DNase (D5025, Sigma-Aldrich), protease inhibitor cocktail (B14001, Bimake, Houston, TX, USA), phosphatase inhibitor cocktail (B15001, Bimake), cell lysis buffer for Western or IP (P0013, Beyotime Biotechnology), protein A/G Dynabeads (B23201, Bimake), DAB Chromogen kit (BDB2004, Biocare, Pacheco, CA, USA), Lipofectamine 3000 (L3000-075, Life Technologies, Waltham, MA, USA), Puromycin (ant-pr-1, Invivogen, Waltham, MA, USA), Tween80(S6702,Selleck), PEG300(S6704, Selleck), Matrigel (354234, Corning, California, USA), D-Luciferin (LUCK-1G, Gold Biotechnology, St Louis, MO).

### Western blotting analysis and immunoprecipitation

Proteins were extracted from tissues and cells using Radioimmunoprecipitation assay (RIPA) lysis buffer (P0013B Beyotime Biotechnology, Jiangsu, China) with 1× phosphatase inhibitor and protease inhibitor (B15001 and B14001, both Bimake, Houston, TX, USA). After quantification utilizing bicinchoninic acid (BCA)(P0012 Beyotime Biotechnology), protein samples were subjected to SDS–PAGE, followed by electrotransfer to polyvinylidene fluoride (PVDF) membranes (Millipore, Billerica, MA, USA). 5% skim milk was used to block the membranes for 1 h at room temperature, followed by incubation with the specific primary antibodies overnight at 4 °C. Thereafter, the membrane was washed three times for 10 min each using Tris-buffered saline-Tween 20 (TBST), and then incubated with HRP-conjugated secondary antibodies for 4 h at 4 °C. For immunoprecipitation (IP), IP/Western lysing solution (P0013, Beyotime Biotechnology) was used to lyse the cells and then IP was performed according to the manufacturer’s instructions followed western blotting detection experiments. The quantitative results of western blotting were obtained using ImageJ 1.8.0 software (NIH, Bethesda, MD, USA).

### Animals and tumor models

The Model Animal Research Center of Nanjing University provided the Balb/c nude mice and C57Bl/6 J mice (all males, 6 weeks old), which were housed under specific-pathogen-free (SPF) conditions in the Experimental Animal Center, the First Affiliated Hospital, School of Medicine, Zhejiang University. Sample sizes were determined based on our and other researchers’ experience with the cell lines used [12, 23, 24], and no samples or animals were excluded. Animals were randomly allocated to each group. During data collection and analysis, two independent investigators were blinded to the group assignment. The first investigator prepared the drugs and labeled them with ABCD names, and then the second investigator administered the drugs and subsequent measurements for analysis according to the ABCD serial numbers. KPC cells (stable clones expressing a short hairpin RNA targeting *Usp8* (shUSP8) and a control plasmid) were injected subcutaneously into the right flanks of the C57Bl/6 J and nude mice (*n* = 7, 5 × 10^5^ cells/mouse). The tumor size was measured using calipers and recorded. Mice were sacrificed after three weeks and tumors were removed and subjected to flow cytometry, western blotting analysis, and immunohistochemistry (IHC) staining. For the subcutaneous tumorigenesis experiments, KPC cells were injected separately into the right flanks of C57Bl/6 J mice and nude mice that had been pretreated or not pretreated with a USP8 inhibitor (1 μM) for 24 h (*n* = 10, 1 × 10^5^ cells/mouse). To assess survival, KPC parental cells and KPC cells with *Usp8* knockdown (KPC-*Usp8* KD) were resuspended in 25 μL of medium added with 12.5 μL of Matrigel and then injected orthotopically into the pancreas of the mice (*n* = 10, 5 × 10^5^ cells/mouse). For each mouse, the time of death was noted. For the subcutaneous tumors used for combination therapy, KPC cells (5 × 10^5^) were resuspended in 50 μL of serum-free Dulbecco’s modified Eagle’s medium (DMEM) and injected subcutaneously injected into the right flank of C57Bl/6 J mice at 6–8 weeks old. When the diameter of the tumors reached 50–100 mm [3], treatment was initiated. The mice were divided randomly into four groups (*n* = 5 per group) comprising the USP8 inhibitor (100 μg per mouse) treatment group, the anti-PD-L1 (αPD-L1) therapy group (200 μg per mouse), the USP8 inhibitor-αPD-L1 combination therapy group, and an untreated control group. Treatments were administered three times weekly for 2 weeks. Calipers were used to measure the tumor size with the formula: volume = (length × width [2]) /2. The mice were killed humanely when the experiment was completed, and the tumors were harvested for subsequent experiments. Combination therapy was also performed using an orthotopic model. Briefly, the pancreas was located in front of the spleen, which was exposed by a small incision on the left abdomen. KPC-Luci cells (5 × 10^5^) were collected and resuspended in 25 μL of medium added with 12.5 μL of Matrigel followed by pancreatic injection using a sterile insulin needle. Treatment started on day 10 and the tumor size was measured using In Vivo Imaging via intraperitoneal injection of 100 mg/kg D-Luciferin (Gold Biotechnology, St Louis, MO, USA) every five days. Mice were divided randomly into four groups (*n* = 5) comprising the USP8 inhibitor (100 μg per mouse) treatment group, the αPD-L1 therapy group (200 μg per mouse), the USP8 inhibitor-αPD-L1 combination therapy group, and an untreated control group. Treatments were administered three times weekly. The mice were sacrificed when the experiment was completed, and the tumors were harvested for subsequent experiments.

### In vivo antibody depletion of CD8 + T cells

CD8 + T cells were depleted by intraperitoneal injection (i.p.) of anti-CD8 monoclonal antibodies (mAbs) (BP0061, Bio X Cell, 200 μg per mouse) 3 days in advance of KPC tumor cell (5 × 10^5^) orthotopic inoculation. αCD8 was given every three days throughout the experiment.

### Cell culture

A *Kras*^*LSL-G12D*^; *Trp53*^*LSL-R172H*^; *Pdx1-Cre* spontaneous tumor mouse model was used to derive the KPC cell line. KPC cells were a gift from Prof. Raghu Kalluri’s laboratory (MD Anderson Cancer Center, Houston, TX, USA). McCoy’s 5 A (Modified) Medium (Thermo Fisher Scientific, Waltham, MA, USA) was used to culture the KPC cells. The ATCC (American Type Culture Collection, Manassas, MD, USA) provided all other PDAC cell lines, which were maintained in Roswell Park Memorial Institute (RPMI)-1640 medium (SH30027.0, GE Healthcare Life Sciences, Chicago, IL, USA) containing 10% fetal bovine serum (FBS) (04-001-1, Biological Industries, Kibbutz Beit-Haemek, Israel) and 1% Penicillin/Streptomycin (CR-15140, Cienry, Zhejiang, China). Short tandem repeat analysis was used to authenticate all the cell lines. For cell culture experiments, individual wells were randomly assigned to treatments. All cultures were routinely evaluated for the presence of mycoplasma contamination using PCR analysis.

### Patient samples

The Department of Hepatobiliary and Pancreatic Surgery, the First Affiliated Hospital, School of Medicine, Zhejiang University provided the paired human pancreatic cancer tissue samples. Wuhan Service bio Technology (Wuhan, China) helped with the preparation of the tumor tissue microarrays used to evaluate USP8 expression from 156 patients with pancreatic cancer. Written informed consent was provided by all the patients. The protocol received approval from the Institutional Review Board at the First Affiliated Hospital, School of Medicine, Zhejiang University. (approval number (2021) IIT (547)).

### Cell migration and invasion assays

Transwell plates with 8-μm pore membranes (353097, Corning Life Sciences, Corning, NY, USA) were used to analyze cell migration and invasion. For the migration assay, Pancreatic tumor cells (KPC and BxPC-3: 5 × 10^5^/well) in serum‐free medium were added to the upper chamber. In total, 500 μL of complete medium containing 10% FBS was added into the lower chamber. DMSO and the USP8 inhibitor (1 μM) were added to the upper chamber after cells attached. After incubation for 24 h, the migratory cells were formalin-fixed and crystal violet (0.1%) stained. Image-Pro Plus 6.0 image analysis software (Media Cybernetics, Bethesda, MA, USA) was used to count the migratory cells. For assays of cell invasion, the Transwell chambers were pre‐coated with Matrigel (1 mg/ml Matrigel matrix, BD Biosciences, Franklin Lakes, NJ, USA). The remaining steps were similar to those described in the migration assay.

### GST pull-down assay

A Pierce GST Protein Interaction Pull-Down Kit purchased from Thermo Scientific (cat. No. 21516) and recombinant proteins including a human USP8-6*His fusion protein (Ag27104, Proteintech), a human CD274-GST fusion protein (Ag12432, Proteintech), and recombinant human GST (ab70456, Abcam), were used to carry out the GST pull-down assay following the producer’s protocol.

### HA-Ub-VS labeling

HA-Ub-VS experiment was carried out as described previously [25]. BxPC-3 cells were lysed using lysis buffer (50 mM Tris pH 7.2, 0.5 mM EDTA, 5 mM MgCl2, 1 mM TCEP, 10% glycerol, protease, and phosphatase inhibitors) on ice. After centrifugation, the lysate (50 μg) was then incubated with DUB-IN-2 (1 μM) or DMSO for 4 h. 2 μM HA-Ub-VS (U-212-025, R&D Systems, Minneapolis, MN, USA) was then added to the lysate and incubated at 37 °C for 70 min, followed by quenching with 4× SDS sample buffer and heating. Proteins were then processed using SDS–PAGE and detected using the indicated antibodies.

### Quantitative real-time reverse transcription PCR (qRT-PCR)

The Trizol LS Reagent (Invitrogen, Carlsbad, CA, USA) was used to isolate total RNA from cells and tissues. A Thermo Scientific™ NanoDrop™ One instrument was then used to determine the RNA concentration. The extracted RNA was then reverse transcribed into cDNA employing a PrimeScript RT reagent kit (RR047A, Takara, Dalian, China). The quantitative real-time PCR step was then carried out using an Applied Biosystems 7500 Fast Real-Time PCR System, Applied Biosystems, (Foster City, CA, USA), with the cDNA as the template, in a 20 μL reaction volume. Relative gene expression was calculated using the standard 2 − ΔΔCt method and was normalized to that of the reference control *Actb* (encoding β-Actin). The efficiency of the primers was tested [26]. They were as follows: human *CD274* (Forward: TTGCTGAACGCCCCATACAA, Reverse: CTGTCCCGTTCCAACACTGA); mouse *Cd274* (Forward: GTCACTTGCTACGGGCGTTTA, Reverse: CGCACCACCGTAGCTGATTA); human *ACTB* (Forward: CTCGCCTTTGCCGATCC, Reverse: TCTCCATGTCGTCCCAGTTG); mouse *Actb* (Forward: CCACCATGTACCCAGGCATT, Reverse: AGGGTGTAAAACGCAGCTCA), all synthesized by Sunya Biotech (Hangzhou, China).

### Histopathological analyses and Immunohistochemistry (IHC) staining

Patient tissue samples and mouse tumor tissues were fixed in neutral buffered formalin (10%), paraffin-embedded, and sectioned at 4 μm thickness. For IHC, the samples were incubated with the following primary antibodies at 4 °C overnight: anti-USP8 (27791-1-AP, Proteintech), anti-PD-L1 (66248-1-Ig, Proteintech), anti-CD8a (98941, Cell Signaling Technology), anti-Granzyme B (44153 S, Cell Signaling Technology), anti-cleaved caspase-3 (9661, Cell Signaling Technology), and anti-Ki67 (12202, Cell Signaling Technology). The next day, the sections were incubated with secondary antibodies conjugated to horseradish peroxidase (HRP). The samples were visualized using a diaminobenzidine (DAB) chromogen kit (BDB2004, Biocare, Pacheco, CA, USA). Image Scope software (Leica Biosystems, Wetzlar, Germany) was used to capture representative images of the tumors. The quantitative results for IHC staining were obtained using ImageJ software and GraphPad Prism 8 (GraphPad Software, San Diego, CA, USA). Microarray slides of PDAC tissue were immunostained using antibodies against USP8 (27791-1-AP, Proteintech) and CD274 (ab205921, Abcam). 3DHISTECH QuantCenter 2.1 software (3Dhistech, Budapest, Hungary) was used to quantify the IHC results.

### Immunofluorescence staining

Immunofluorescence (IF) staining was performed to identify the location of USP8 and CD274 in tumor cells and tissues. Cold 4% polyoxymethylene was used to fix the cells for 10 min following blocking in 3% BSA. The cells were then incubated at 4 °C overnight with primary antibodies targeting USP8 (ab228572, Abcam) and CD274 (14-5982-82, Thermo Scientific), followed by secondary antibody incubation. For IF of tissues, most of the steps were similar to those for IHC. Fluorescent images of the cells and tissues were acquired using a TCS SP8 X confocal microscope (Leica).

### Cell transfections

When KPC and BxPC-3 cells reached 70% confluence, they were stably transfected with lentiviral particles encoding mouse or human USP8. The experiment was performed following to the supplier’s protocol. Post-transfection, the cells were incubated for 24 h and selected using 10 μg/mL puromycin for 1 week.

### Flow cytometry analysis

Mouse tumors were mechanically dissociated and digested in RPMI-1640 supplemented with 2% FBS, DNase (10 μg/mL) (D5025, Sigma-Aldrich, St. Louis, MO, USA), collagenase IV (1 mg/mL) (17104019, Thermo Fisher Scientific), and CaCl_2_ (3 mM) (21115, Sigma-Aldrich) at 37 °C for 1 h. The dissociated tissues were filtered through 70 mm strainers to obtain single cells. Then, to remove any non-immune cells, the cells were then washed and resuspended in a 36% Percoll solution. The following antibodies were then used to stain the cells for flow cytometric analyses: anti-CD326, anti-CD274, anti-CD45, anti-CD3, anti-CD4, anti-CD8, anti-Granzyme B, anti-IFN-r, anti-TNF-a, and anti-Perforin. A Beckman CytoFLEX LX instrument was used for flow cytometry and FlowJo software (FlowJo LLC, Ashland, OR, USA) was used to analyze the data.

### T cell-mediated tumor cell killing

A CD8 + T cell isolation kit (130-104-075, Miltenyi Biotec, Cologne, Germany) was used to isolate CD8 + T cells from mouse spleens, which were activated with 3.5 μg/ml CD3 antibody (100359, BioLegend, San Diego, CA, USA) and 1 μg/ml CD28 antibody (102116, BioLegend) for 48 h. KPC cells were treated or untreated with USP8 inhibitor (1 μM, 24 h). Parental and *Usp*8 KD KPC cells were incubated overnight. KPC cells were treated with the USP8 inhibitor (1 μM, 24 h) alone or combined with αPD-L1 (10 μg/ml, 24 h). Then, isolated activated CD8^+^ T cells were added to KPC cells at a ratio of 4:1 and co-incubated for 48 h. Tumor cells were stained using crystal violet (0.5%) and measured spectrophotometrically at OD 570 nm.

### Study of safety

The safety study of DUB-IN-2 (USP8 inhibitor) and anti-PD-L1 (αPD-L1) in vivo was conducted on C57Bl/6 J mice. DUB-IN-2 (100 μg per mouse) and αPD-L1 (200 μg per mouse) were administered three times weekly for 2 weeks. Bodyweight was recorded every three days. Serum samples were separated from the blood for biochemical testing including ALT (alanine transaminase), CREA (creatinine), UREA (urea), UA (uric acid), ALB (albumin), and TP (Total Protein) by Wuhan Service bio Technology (Wuhan, China).

### Bioinformatic analyses

The *CD274* and *USP8* gene expression data across multiple tumor samples and normal samples were acquired from The Cancer Genome Atlas (TCGA) database and the Genotype-Tissue Expression (GTEx) database. Sample types and sources were critically scrutinized and analyzed using the R software.

### Statistical analyses

Means ± SD of at least three independent biological replicates is used to present the statistical results. Differences between the two groups were determined utilizing Student’s *t* test, and differences among multiple groups were determined utilizing one-way analysis of variance (ANOVA). The Kaplan-Meier method was used to analyze overall survival curves, which were compared using a log-rank test. Correlations between two variables were assessed utilizing Spearman’s rank correlation. These analyses were performed using SPSS (V20, IBM Corp., Armonk, NY, USA) and GraphPad Prism 8.0 software. Statistical significance was accepted at *p* < 0.05 (in the figures, * indicates *p* < 0.05, ** indicates *p* < 0.01, *** indicates *p* < 0.001, and **** indicates *p* < 0.0001).

## Results

### USP8 is highly expressed in pancreatic cancer

**Fig. 1:**
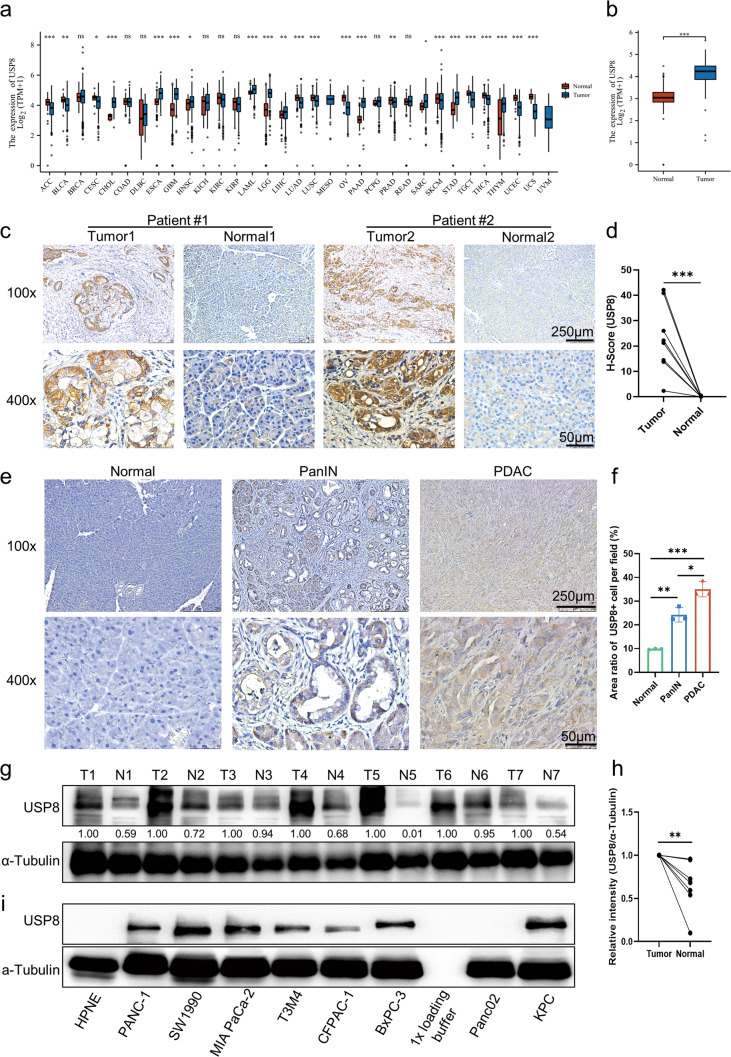
USP8 is highly expressed in pancreatic cancer. **a** The *USP8* mRNA expression profile between tumor samples from the TCGA database and normal tissues from the TCGA and GTEx database in pan-cancer. Blue codes represent tumors in which *USP8* is expressed in tumor samples higher than normal tissues. **b** Relative *USP8* mRNA expression in pancreatic adenocarcinoma (PDAC) tissues (*n* = 179) and normal pancreatic tissues (*n* = 171) were included based on the TCGA and GTEx database. **c** USP8 levels as measured using IHC staining in pancreatic tumor tissues and paired normal tissues from eight patients, **d** statistical analysis of the results in (**c**) (Tumor: Pancreatic tumor tissue; Normal: Normal pancreatic tissue). Scale bars =100×: 250 μm and 400×: 50 μm. **e** Expression of USP8 in KPC mice pancreatic tumor tissues, PanIN1 lesions, and normal mouse pancreas tissues determined using IHC staining in three mice, (**f**) statistical analysis of the results in (**e**). Scale bars = Scale bars =100×: 250 μm and 400×: 50 μm. **g** Western blotting analysis of USP8 levels in clinical pancreatic tissue samples from seven patients, **h** statistical analysis of the results in (**g**) (T: Pancreatic tumor tissue; N: Normal pancreatic tissue). **i** Analysis of USP8 protein levels among normal pancreatic ductal cells (HPNE), human pancreatic cancer cell lines (BxPC-3, CFPAC-1, T3M4, MIA PaCa-2, SW1990, and PANC-1), and mouse pancreatic cancer cell lines (Panc02, KPC) using western blotting. In (**b**), data are shown as boxplots in which the median is shown as the middle line; the first and third quartiles correspond to lower and upper hinges; the upper whisker extends from the hinge to the largest value by no more than 1.5× the inter-quartile range (IQR) from the hinge; the lower whisker extends from the hinge to the smallest value at most 1.5× IQR of the hinge, while data appearing beyond the end of the whiskers represent individually plotted outlying points. The results are shown as the means ± SD representative experiments in (**f**). The data represent three independent experiments. *p < 0.05, **p < 0.01, ***p < 0.001 assessed via a two-tailed *t* test; ns: not significant.

We performed bioinformatic analysis of *USP8* using mRNA expression data from tumor samples and paired normal tissues at the TCGA (Fig. 1a). The analysis revealed that the pancreatic cancer samples had a high expression of *USP8* compared with that in paired normal tissues (Fig. 1b). To further validate the differential expression of USP8, we compared the USP8 levels in the paired pancreatic tumor tissue samples from patients using IHC staining, which showed significantly higher USP8 levels in PDAC tissues than in normal tissues (Fig. 1c, d). Compared with that in normal mouse pancreas tissues, IHC also demonstrated elevated expression of USP8 in KPC (*Kras*^*LSL-G12D*^; *Trp53*^*LSL-R172H*^; *Pdx1*-Cre) tumor tissues, which had been initially activated in PanIN1 lesions (Fig. 1e, f). Western blotting confirmed the significantly higher USP8 protein levels in clinical pancreatic tumor tissues relative to those in matched para-cancerous tissues (Fig. 1g, h). Besides, western blotting was used to assess the level of USP8 in pancreatic cancer cell lines. As expected, high levels of USP8 were detected in human pancreatic cancer cell lines (BxPC-3, CFPAC-1, T3M4, MIA PaCa-2, SW1990, and PANC-1) and the mouse pancreatic cancer cell line (KPC), but not in Panc02; no USP8 was detected in HPNE cells (normal pancreatic ductal cells) (Fig. 1i). Through bioinformatic analysis in the TCGA database, we observed high USP8 expression in immune “hot” tumors and low expression in immune “cold” tumors (Fig. S1a). However, after subdividing the immunogenic subtype into six classes, USP8 expression did not differ (Fig. S1b). To verify that USP8 is important in pancreatic cancer, the USP8 level was detected in a PDAC tissue microarray. Overall survival (OS) was not significantly different according to Kaplan-Meier survival analysis (*p* = 0.833) among patients with PDAC with different USP8 protein levels (Fig. S1c). However, USP8 expression was demonstrated to be associated significantly with the tumor-node-metastasis (TNM) stage in a clinical association study in various patient-derived pancreatic cancer cohorts (*p* = 0.041) (Supplementary Table 1). Together, these results indicated that USP8 is highly expressed in pancreatic cancer compared with that in paired normal tissue.

### USP8 deficiency significantly reduces tumor migration and invasion and improves anti-tumor immunogenicity

**Fig. 2:**
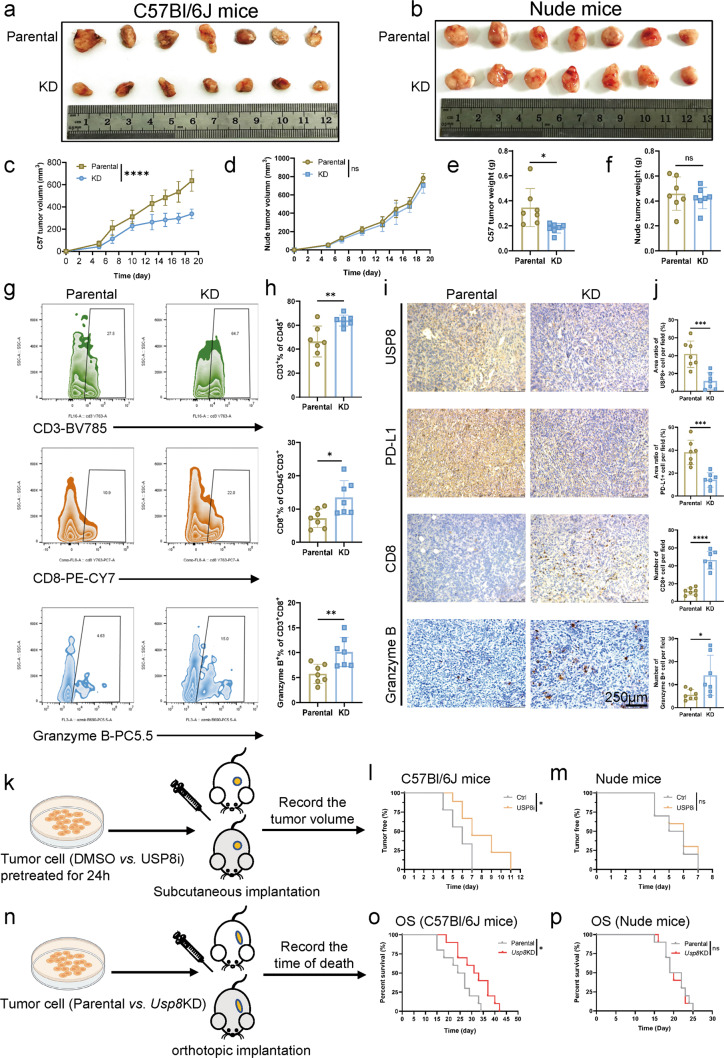
USP8 deficiency significantly improves anti-tumor immunogenicity. **a** Photographs of tumors and growth curves (**c**) in immunocompetent mice. The tumors were measured at the indicated time points before being excised at the end of the experiment (*n* = 7). **b** Photographs of tumors and growth curves (**d**) in immunodeficient mice. The tumors were measured at the indicated time points before being excised at the end of the experiment (*n* = 7). **e**, **f** Tumors weights in the immunocompetent and immunodeficient mice were recorded at the end of the experiment (*n* = 7). **g**, **h** Flow cytometry analysis of tumor-infiltrating lymphocytes (TILs; *n* = 7). **i**, **j** IHC staining and quantification of USP8 expression, PD-L1 expression, and TILs (*n* = 7). Scale bars = 250 μm. **k** Protocols of the pretreatment of pancreatic cancer cells (1 × 10^5^) with DMSO or the USP8-specific inhibitor (1 μM, 24 h). The treated cells were injected subcutaneously into immunodeficient and immunocompetent mice (*n* = 10). **l**, **m** The incidence of tumors in the immunodeficient and immunocompetent mice at the indicated times. **n** Protocol for the separate and orthotopic injection of parental and *Usp8*-depleted pancreatic cancer cells (5 × 10^5^) into immunodeficient and immunocompetent mice (*n* = 10). **o**, **p** Survival of *Usp8*-depleted pancreatic tumor-bearing immunocompetent and immunodeficient mice (*n* = 10). The data in (**l** and **m**) were generated using Kaplan-Meier survival curves based on log-rank tests. The data in (**o** and **p**) were generated using the Gehan–Breslow–Wilcoxon test and the Kaplan-Meier method. The results are shown the means ± SD of representative experiments in (**c**–**h**, and **j**). The data represent three independent experiments. *p < 0.05, **p < 0.01, ***p < 0.001, ****p < 0.0001 assessed via a two-tailed *t* test; ns: not significant.

Transwell assays were used to determine the effects on invasion and migration of USP8. USP8 inhibition significantly reduced KPC and BxPC-3 cell invasion and migration (Fig. S2a–d). Moreover, USP8 inhibition reduced epithelial-to-mesenchymal transition and reduced metastasis, according to western blotting analysis (Fig. S2e, f). To further reveal the important role of USP8, we investigated the connection between *USP8* and immunity-related factors from the TCGA database. The analysis revealed that USP8 expression was associated positively with certain immunosuppressive factors (Fig. S3a–c). We constructed a stable *Usp8* knockdown (KD) KPC cell line using shRNA lentivirus to determine whether and how USP8 deficiency improves anti-tumor immunogenicity. In immunocompetent C57Bl/6 J mice, tumors formed by *Usp8* KD cells (5 ×10^5^) showed decreased tumor size and weight relative to those induced by parental KPC cells (5 ×10^5^); however, this effect was not observed when the experiment was repeated using Nude (immunodeficient) mice (Fig. 2a–f). Analysis using flow cytometry showed that the amount and function of tumor infiltrated activated T-cells increased in *Usp8* KD cell-derived tumors (Fig. 2g, h). Similarly, IHC staining revealed significant expansions of CD8 + cells, Granzyme B + cells, cleaved caspase-3+ cells, and marked reductions of Ki67+ cells in *Usp8* KD cell-derived tumors. Meanwhile, additional IHC staining in *Usp8* KD cell-derived tumors also showed decreased USP8 expression and PD-L1 expression (Fig. 2i, j; Fig. S4a, b). Next, immunocompetent and immunodeficient mice were injected separately and subcutaneously with pancreatic cancer cells pretreated with DMSO or a USP8-specific inhibitor (1 μM, 24 h), to further assess the impact of inhibiting USP8 on pancreatic cancer tumorigenesis and development (Fig. 2k). USP8 inhibitor pretreatment led to reduced tumorigenesis (Fig. 2l) in the immunocompetent mice, but not in the nude mice (Fig. 2m). Besides, parental and *Usp8* KD KPC cells were injected separately and orthotopically into immunodeficient and immunocompetent mice (Fig. 2n). According to the recorded time of death, extended survival was observed in immunocompetent mice with *Usp8* KD tumors compared with the mice injected with parental KPC cells (Fig. 2o); however, the immunodeficient mice did not receive this survival benefit (Fig. 2p). Furthermore, USP8 inhibition and USP8 deficiency significantly improved activated tumor cell killing mediated by T cells in vitro (Fig. S4c–f). Moreover, we constructed a stable *Kras*^*G12D*^ knockdown KPC cell line to determine which molecular PDAC subtype USP8 inhibition would most likely apply to (Fig. S5a). We constructed tumor models and administrated the USP8 inhibitor alone to mice bearing subcutaneous KPC parental cells and *Kras*^*G12D*^ KD cells (Fig. S5b). We observed that the tumor size, tumor volume, and tumor weight in the USP8 inhibitor group decreased significantly compared with those in the control group, and the degree of reduction was similar in the two groups (Fig. S5c–e). Overall, USP8 was linked to the promotion, migration, and invasion of pancreatic tumors. USP8 deficiency could exert anti-tumor effects in an immunity-dependent manner and USP8 inhibition could exert a similar tumor suppressive effect in different molecular subtypes of pancreatic cancer.

### There is a positive interaction between PD-L1 and USP8 in pancreatic cancer

**Fig. 3:**
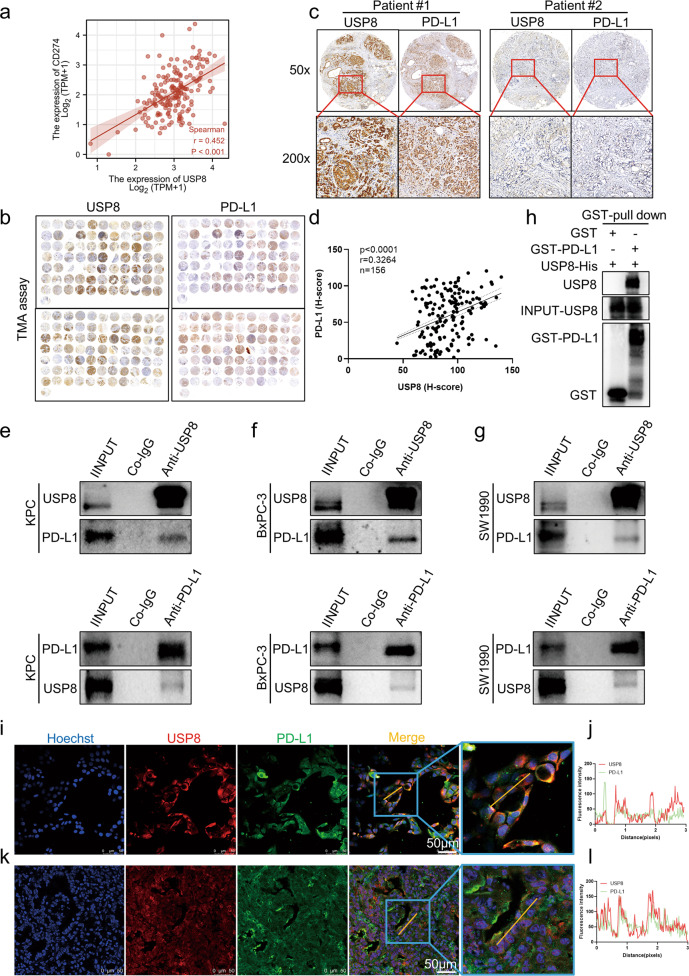
In pancreatic cancer, USP8 interacts positively with PD-L1. **a** Bioinformatic analysis of the correlation between *USP8* and *CD274* mRNA expression in pancreatic cancer samples from the TCGA database (*n* = 179). **b–d** Photographs and statistical analyses of USP8 and PD-L1 in tumor tissue microarrays using IHC (*n* = 156). **e–g** KPC, BxPC-3, and SW1990 pancreatic cancer cell lines were analyzed separately by western blotting with the indicated antibodies. The images are representative of three independent experiments. **h** USP8-His and GST-PD-L1 proteins were subjected to GST-pull down assays. The images are representative of three independent experiments. **i** Photographs of immunofluorescence staining showing the interaction of USP8 and PD-L1 in KPC cells and fluorescence intensity plots (**j**) showing the co-localization of USP8 and PD-L1. The images are representative of three independent experiments. **k** Photographs of immunofluorescence staining showing the interaction of USP8 and PD-L1 in KPC mice pancreatic tumor tissues and fluorescence intensity plots (**l**) showing the co-localization of USP8 and PD-L1. The images are representative of three independent experiments. In (**d**), Spearman correlations and *p* values calculated using Spearman’s test are shown.

The PD-1/PD-L1 inhibitory signal plays an important role in tumor immune escape and is an important target of anti-tumor immunotherapy; therefore, we further assessed whether USP8 interacts with PD-L1. A significant correlation between *USP8* and *PDL1* mRNA expression in pancreatic cancer samples was observed using bioinformatic analysis at the TCGA database (Fig. 3a). In addition, analysis using pancreatic tumor tissue microarrays indicated their correlation at the protein level (Fig. 3b–d). Moreover, we observed that endogenous USP8 and endogenous PD-L1 interacted in pancreatic cancer cell lines (KPC, BxPC-3, SW1990) (Fig. 3e–g). Furthermore, direct binding of USP8 to PD-L1 was demonstrated in vitro using a GST pull-down assay (Fig. 3h). Similarly, immunofluorescence analysis and fluorescence intensity plots showed significant USP8/PD-L1 colocalization (USP8: red; PD-L1: green) in KPC cells, BxPC-3 cells, KPC mouse pancreatic tumor tissues, and human pancreatic tumor tissues (Fig. 3i–l; Fig. S6a–d). These results revealed that USP8 and PD-L1 interact positively in pancreatic cancer.

### Ubiquitination-mediated degradation of PD-L1 by proteasomes is inhibited by USP8

**Fig. 4:**
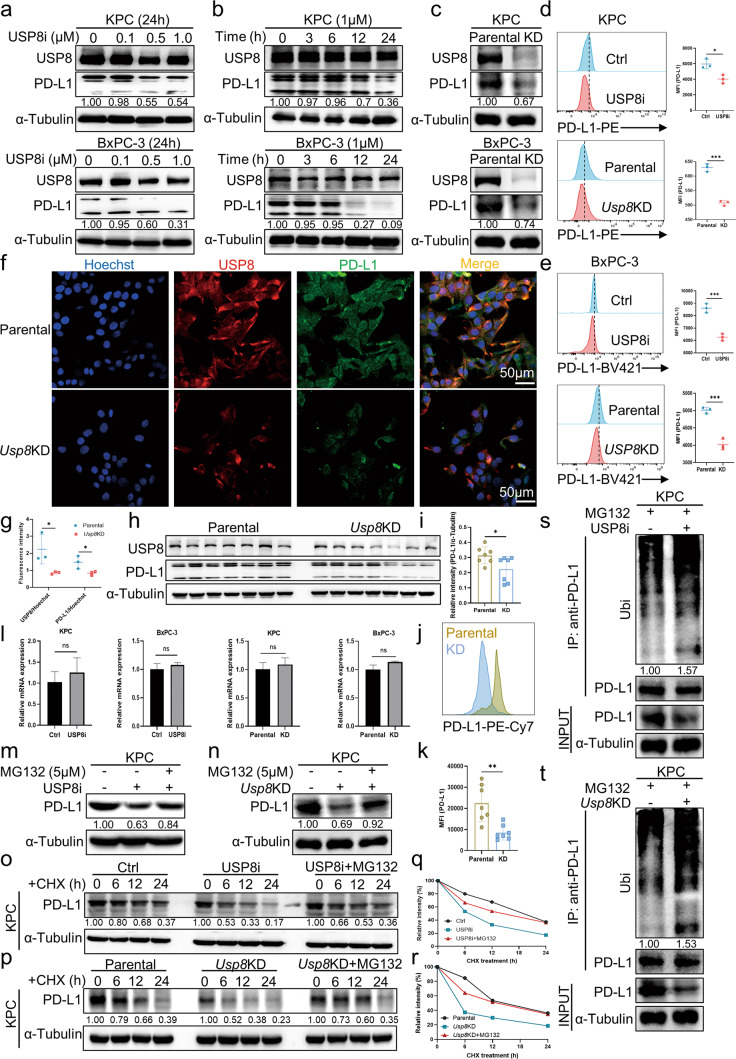
Ubiquitination-mediated degradation of PD-L1 by proteasomes is inhibited by USP8. **a–c** Levels of PD-L1in pancreatic cancer cell lines (KPC, BxPC-3) treated with a concentration gradient and time gradient of the USP8 inhibitor and after *Usp8* knockdown, as assessed using western blotting. The image is representative of three independently performed experiments. **d**, **e** Statistical analysis of the levels of PD-L1 in pancreatic cancer cell lines treated with the USP8 inhibitor (1 μM, 24 h) and *Usp8* KD and subjected to flow cytometry. A representative image of three independent experiments is shown. **f** Photographs of immunofluorescence staining showing USP8 and PD-L1 expression in parental and *Usp8*-depleted KPC cells, and the statistical analysis of the results (**g**). The image is representative of three independently performed experiments. **h–k** Statistical analysis of USP8 and PD-L1 levels in tumor samples, as assessed using flow cytometry and western blotting analyses. **l** Statistical analyses of the qRT-PCR results showing *PDL1* mRNA levels in pancreatic cancer cell lines treated with the USP8 inhibitor (1 μM, 24 h) and *Usp8* knockdown. A representative image of three independent experiments is shown. **m** The level of PD-L1 after USP8 inhibitor (1 μM, 24 h) treatment in KPC cells treated with MG132 (5 μM, 12 h), as assessed using western blotting. A representative image of three independent experiments is shown. **n** The level of PD-L1 in MG132 (5 μM, 12 h)-treated parental and *Usp8* KD KPC cells, as assessed using western blotting. A representative image of three independent experiments is shown. **o** Analysis of PD-L1 stability in cycloheximide (CHX) (200 μg/mL) pretreated KPC cells incubated with the USP8 inhibitor (1 μM, 24 h). A representative image of three independent experiments is shown. **p** Analysis of PD-L1 stability in cycloheximide (CHX) (200 μg/mL) treated parental and *Usp8* KD KPC cells. A representative image of three independent experiments is shown. **q**, **r**, A densitometer was used to quantify the intensity of PD-L1 protein expression and the results are representative of three independent experiments. **s** Assay of PD-L1 ubiquitination in KPC cells. The cells were treated with MG132 (5 μM, 12 h) followed by USP8 inhibitor (1 μM, 24 h) treatment and then western blotting was used to detect PD-L1 and ubiquitin. **t** Assay of PD-L1 ubiquitination in parental and *Usp8* KD KPC cells. Cells were treated with MG132 (5 μM, 12 h) then western blotting was used to detect PD-L1 and ubiquitin. Results are shown as the means ± SD of representative experiments in (**d**, **e**, **g**, **i**, **k**, **l**, **q** and **r**). The data represent three independent experiments. *p < 0.05, **p < 0.01, **p < 0.001 assessed via a two-tailed *t* test; ns: not significant.

Considering that USP8 and PD-L1 correlate positively, there might be a regulatory mechanism between them. The USP8 inhibitor (DUB-IN-2) (1 μM, 4 h) was demonstrated to inhibit USP8 activity well in BxPC-3 cell lysate using the HA-Ub-VS experiment (Fig. S7a). The PD-L1 protein level in pancreatic cancer cell lines was downregulated after treatment with a concentration gradient and time gradient of the USP8 inhibitor and after *Usp8* knockdown, according to western blotting analysis (Fig. 4a–c; Fig. S7b, c). Similarly, treatment using the USP8 inhibitor and *Usp8* knockdown downregulated PD-L1 expression in pancreatic cancer cell lines, according to flow cytometry analysis (Fig. 4d, e). By contrast, *USP8* overexpression upregulated PD-L1 levels (Fig. S7d, e). Statistical analysis of the immunofluorescence staining results showed that USP8 (red) and PD-L1 (green) levels were downregulated in *Usp8*-depleted KPC cells in comparison with those in parental KPC cells (Fig. 4f, g). Furthermore, PD-L1 expression was downregulated significantly in *Usp8* KD pancreatic tumors compared with that in parental KPC tumors (Fig. 4h–k). However, according to the qRT-PCR results, there was no significant change in *PDL1* mRNA levels after treatment with the USP8 inhibitor or *Usp8* knockdown, which illustrated that USP8 regulation of PD-L1 occurs at the post-translational level rather than at the transcriptional level (Fig. 4l). Thus, we hypothesized that USP8 regulates PD-L1 levels via PTM in pancreatic cancer. Interestingly, PD-L1 downregulation mediated by USP8 inhibition or depletion was restored after treatment with the proteasome inhibitor MG132 in pancreatic cancer cells, suggesting that USP8 regulates PD-L1 via the proteasome pathway (Fig. 4m, n; Fig. S7f, g). To verify that USP8 regulates PD-L1 stability, we performed a half-life analysis, which showed that PD-L1 had a shortened half-life and was degraded rapidly in both USP8 inhibited and depleted cells, a reduction that could be rescued by MG132 (Fig. 4o-r; Fig. S7h–k). Subsequently, we assessed the effect of USP8 inhibition and depletion on PD-L1 ubiquitination. The results indicated that PD-L1 ubiquitination increased markedly in the USP8 inhibited and depleted cells compared with that in the control cells (Fig. 4s, t; Fig. S7l, m). Besides, EGFR levels were not affected by USP8 inhibition or depletion in vivo and in vitro (Fig. S8a-e). Therefore, we determined that USP8 could decrease PD-L1 degradation by reducing the ubiquitination level of PD-L1. Collectively, these findings showed that USP8 stabilizes PD-L1 levels in pancreatic cancer cells by inhibiting the degradation of PD-L1 via the ubiquitin-proteasome pathway.

### The combination of the USP8 inhibitor and USP8 deficiency with αPD-L1 effectively suppresses pancreatic tumor growth and activates cytotoxic T-lymphocytes

**Fig. 5:**
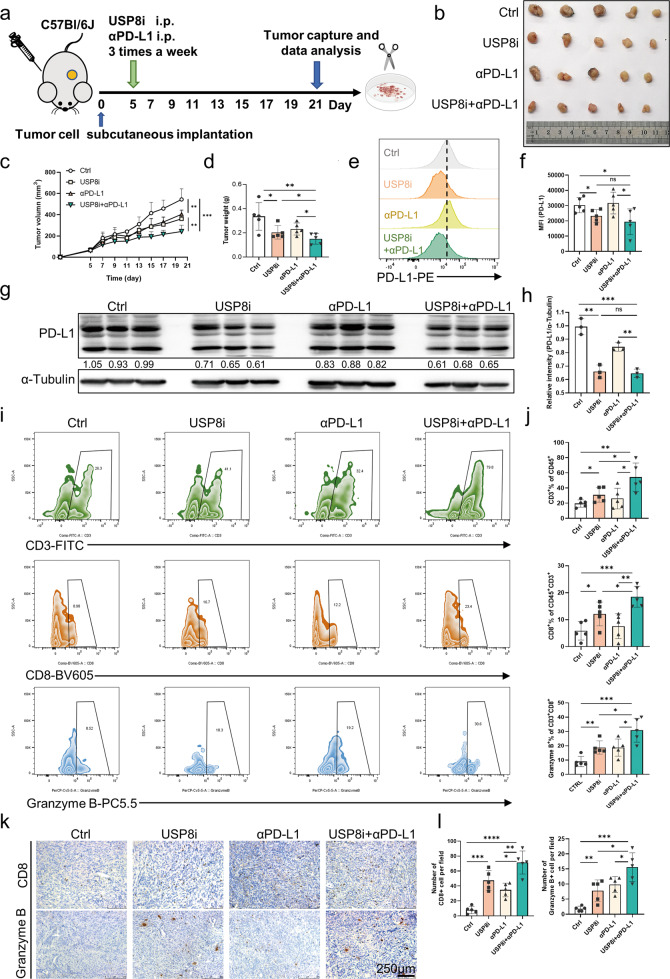
Pancreatic tumor growth in vivo is suppressed effectively using a USP8 inhibitor combined with αPD-L1. **a** Protocol schematic of the combination of USP8 inhibitor and αPD-L1 therapy for mice implanted subcutaneously with KPC cells (5 × 10^5^). **b** Photographs of tumors removed from mice treated with the USP8 inhibitor, αPD-L1, or their combination (*n* = 5). **c** Curves showing the tumor growth in mice treated with the USP8 inhibitor (100 μg/mouse), αPD-L1 (200 μg/mouse), or their combination (*n* = 5). **d** The statistical plot of tumor weights of the four groups (*n* = 5). **e**, **g** PD-L1expression assessed using flow cytometry and western blotting in a subcutaneous tumor model, statistical results (**f**, **h**) shown. **i**, **j** Tumor-infiltrating lymphocytes (TILs) assessed using flow cytometry and the statistical analysis of the results (*n* = 5). **k**, **l** Representative images of IHC staining and quantification of TILs (*n* = 5). Scale bars = 250 μm. The results are shown as means ± SD from representative experiments in (**c**, **d**, **f**, **h**, **j** and **l**). The data represent three independent experiments. *p < 0.05, **p < 0.01, ***p < 0.001, ****p < 0.0001 assessed via a two-tailed *t* test; ns: not significant.

To explore the anti-tumor role of the USP8 inhibitor via regulation of PD-L1 levels in vivo, we constructed tumor models and administrated the USP8 inhibitor alone or combined with αPD-L1 to mice bearing subcutaneous KPC-derived tumors (Fig. 5a). We observed that in the USP8 inhibitor group, the tumor size, tumor volume, and tumor weight decreased significantly compared with those in the control group, and these reductions were more significant in the combination group (Fig. 5b–d). Additionally, the bodyweight of the mice and the relative mouse bodyweight on the last day showed a healthy and constant gain (Fig. S9a, b), and there were no obvious changes in spleen weights among the four groups (Fig. S9c, d). We further performed blood biochemical tests including ALT, CREA, UREA, UA, ALB, and TP to evaluate the safety of the drugs. The results showed that there was no significant difference for each indicator among the four groups (Fig. S9e). Compared with the control group, the USP8 inhibitor decreased the PD-L1 level significantly according to western blotting and flow cytometry analyses (Fig. 5e–h). Moreover, flow cytometric analysis and IHC staining revealed that cotreatment with αPD-L1 significantly increased the number and activation of tumor-infiltrating cytotoxic CD8 + T cells in the mice (Fig. 5i-l). Additional IHC staining also showed significant expansions of cleaved caspase-3+ cells, marked reductions in Ki67+ cells, and decreased PD-L1 levels in the combination group compared with those in the control group (Fig. S9f, g). Furthermore, in vitro, the USP8 inhibitor-αPD-L1 combination therapy promoted activated T cell-mediated tumor cell killing significantly compared with the USP8 inhibitor treatment group or the αPD-L1 therapy group (Fig. S9h, i). Taken together, these results suggested that combining the USP8 inhibitor with PD-L1 blockade could enhance anti-tumor immunity and suppress the growth of pancreatic tumors.

**Fig. 6:**
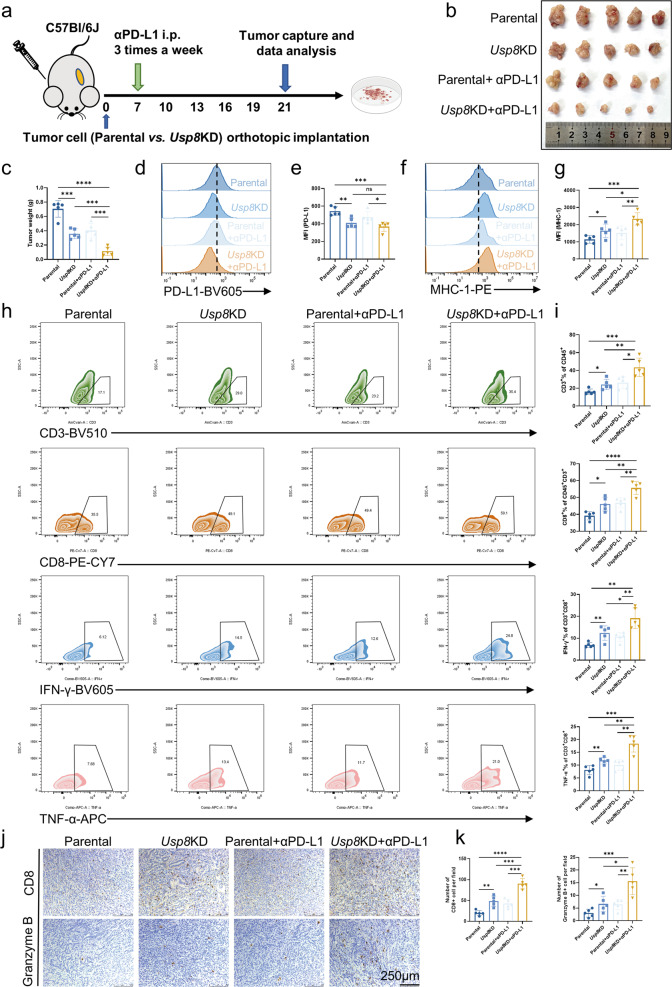
USP8 deficiency combined with αPD-L1 promotes antitumor immunity. **a** Schematic of the protocol of USP8 deficiency and αPD-L1 combination therapy for orthotopic KPC parental and *Usp8* KD cell (5 × 10^5^)-bearing mice. **b** Photographs of tumors removed from the mice of each group (*n* = 5). **c** The statistical plot of tumor weights of the four groups (*n* = 5). **d** Flow cytometry of PD-L1 levels in an orthotopic tumor model, statistical results (**e**) are shown. **f** Flow cytometry of MHC-1 levels in an orthotopic tumor model, statistical results (**g**) are shown. **h**, **i** Flow cytometry of CD3 + T cells, CD8 + T cells, IFN-γ + CD8 + T cells, TNF-α + CD8 + T cells in the tumor region and the statistical analysis of the results (*n* = 5). **j**, **k** Representative images of IHC staining and quantification of TILs (*n* = 5). Scale bars = 250 μm. The results are displayed as the means ± SD from representative experiments in (**c**, **e**, **g**, **i** and **k**). The data represent three independent experiments. *p < 0.05, **p < 0.01, ***p < 0.001, ****p < 0.0001 assessed via a two-tailed *t* test; ns not significant.

To further investigate USP8’s role in the immune responses of pancreatic cancer, we constructed orthotopic parental and *Usp8* KD KPC cell-bearing mice combined with αPD-L1 (Fig. 6a). The best tumor suppressor effect was achieved by *Usp8* KD combined with αPD-L1 (Fig. 6b, c). Flow cytometry showed that USP8 deficiency reduced PD-L1 expression and increased MHC-1 expression, which was also confirmed in vitro (Fig. 6d–g; Fig. S10a, b). Importantly, the combined therapy enhanced the population of CD3 + T cells, CD8 + T cells, IFN-γ + CD8 + T cells, and TNF-α + CD8 + T cells markedly in the tumor region (Fig. 6h, i). Moreover, IHC staining showed similar results to those in the subcutaneous model administrated with USP8 inhibitor and αPD-L1(Fig. 6j, k; Fig. S10c, d).

**Fig. 7:**
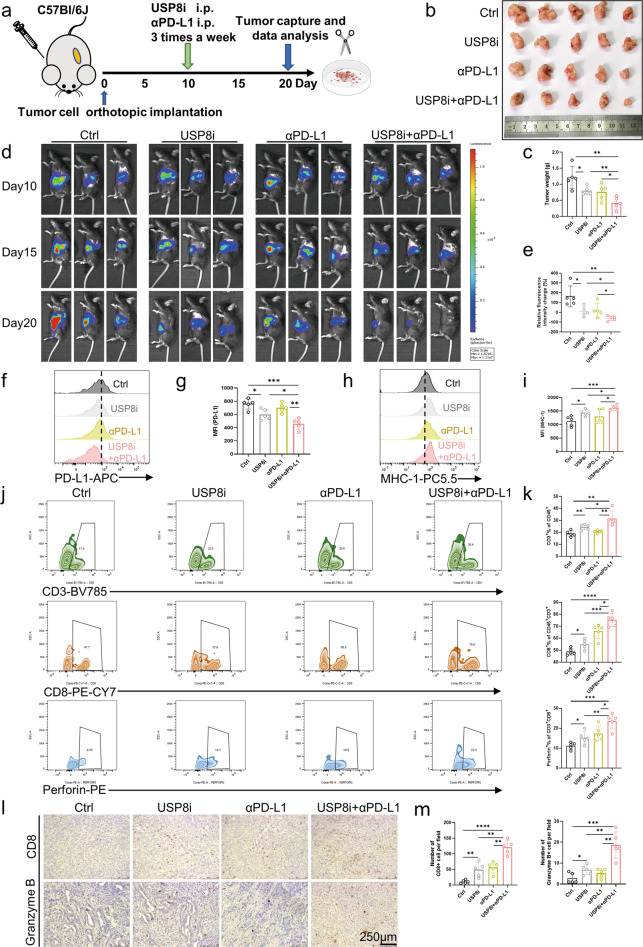
USP8 inhibitor sensitizes pancreatic cancer to immunotherapy targeting PD-L1. **a** Schematic diagram of the protocol for USP8 inhibitor and αPD-L1 combination therapy for orthotopic KPC-Luci cell (5 × 10^5^)-bearing mice. **b** Photographs of tumors removed from mice treated with the USP8 inhibitor, αPD-L1, or their combination (*n* = 5). **c** The statistical graph of tumor weights of the four groups (*n* = 5). **d** Representative images displaying changes in the luminescence intensity of tumors obtained using In Vivo Imaging and the relative luminescence intensity change (**e**) on the last day. **f** Flow cytometry of PD-L1 levels in an orthotopic tumor model, statistical results (**g**) are shown. **h** Flow cytometry of MHC-1 levels in an orthotopic tumor model, statistical results (**i**) are shown. **j, k** Tumor-infiltrating lymphocytes (TILs) assessed using flow cytometry and the statistical analysis of the results (*n* = 5). **l**, **m** Representative images of IHC staining and quantification of TILs (*n* = 5). Scale bars = 250 μm. The results are shown as the means ± SD from representative experiments in (**c**, **e**, **g**, **i**, **k** and **m**). The data represent three independent experiments. *p < 0.05, **p < 0.01, ***p < 0.001, ****p < 0.0001 assessed via a two-tailed *t* test; ns not significant.

To verify the immunotherapeutic mechanisms of combining the USP8 inhibitor and αPD-L1, we constructed orthotopic KPC-Luci cell-bearing mice (Fig. 7a). As expected, compared with the control group, the most pronounced decrease in tumor size and weight was observed in the co-administration group (Fig. 7b, c). Through comparing changes in the luminescence intensity of tumors using In Vivo Imaging and the relative luminescence intensity change on the last day, we found that the tumors shrank gradually according to the duration of drug administration, with the most pronounced reduction in the combination group (Fig. 7d, e). Additionally, the application of the USP8 inhibitor increased the expression of MHC-1, according to flow cytometry analyses (Fig. 7h, i). Meanwhile, other experimental results showed similar results to those in the subcutaneous model administrated with USP8 inhibitor and αPD-L1 (Fig. 7f, g, j–m; Fig. S11a-h). Moreover, since CTLA-4 is another important immune checkpoint, we compared the USP8 inhibitor in combination with αCTLA-4 with the USP8 inhibitor in combination with αPD-L1. (Fig. S12a). We observed that the USP8 inhibitor combined with αCTLA-4 group showed reductions compared with the control group in terms of tumor size and tumor weight, but these reductions were more significant in the USP8 inhibitor and αPD-L1 combination group (Fig. S12b, c). Flow cytometry analysis revealed that cotreatment with αPD-L1 could better activate immunity than cotreatment with αCTLA-4 (Fig. S12d–i). Interestingly, we observed that the animal model did not lead to liver and lung tumor metastasis, but the number of spleens with tumor migration and invasion decreased after treatment with the USP8 inhibitor and αPD-L1; however, there was no change in abdominal lymph nodes (Fig. S13a–f). Therefore, the combination of USP8 inhibitor and αPD-L1 could reduce tumor migration in vivo. Collectively, the above results verified that USP8 inhibition activates cytotoxic T-lymphocytes by regulating PD-L1 stability.

### The anti-tumor immunity induced by the combination of USP8 inhibitor and αPD-L1 is dependent on the PD-L1 pathway and CD8 + T cells

**Fig. 8:**
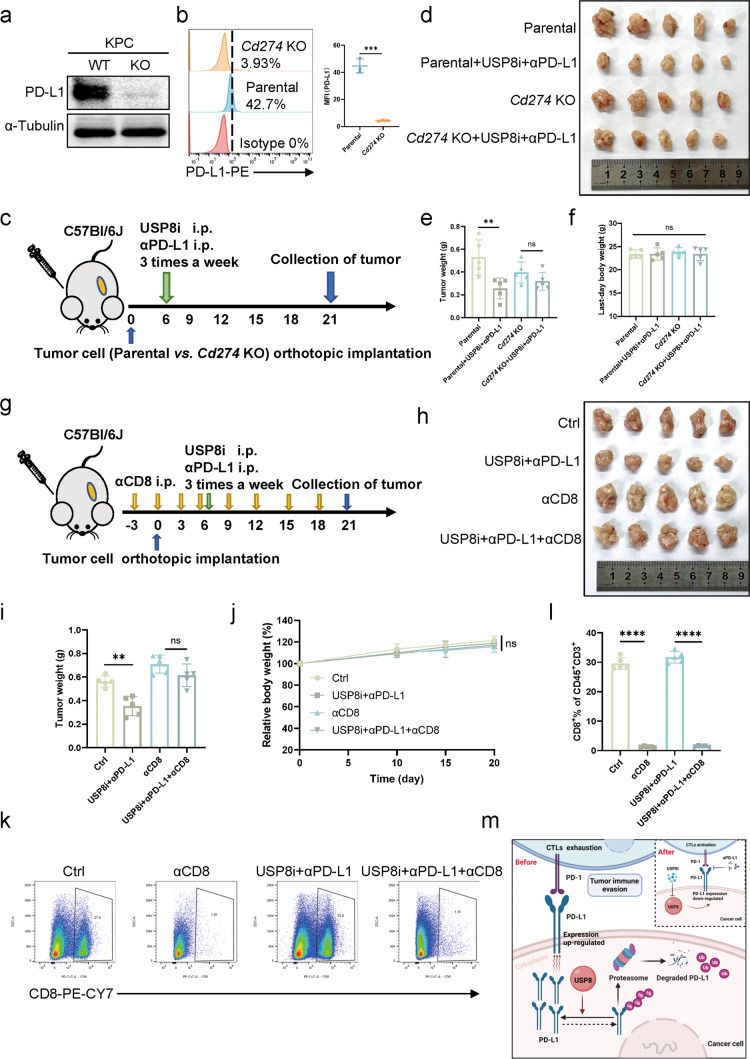
The anti-tumor immunity induced by the combination therapy depends on the PD-L1 pathway and CD8 + T cells. **a**, **b** Western blotting and flow cytometry results validating *Cd274* KO in KPC cell line. **c** Schematic of the protocol for USP8 inhibitor and αPD-L1 combination therapy for orthotopic KPC parental and *Cd274* KO cell (5 ×10^5^)-bearing mice. **d** Photographs of tumors removed from mice of each group (*n* = 5). **e** The statistical plot of tumor weights of the four groups (*n* = 5). **f** The body weight of mice on the last day (*n* = 5). **g** Experimental design for CD8 + T cells depletion in orthotopic KPC (5 × 105)-bearing mice receiving the combination therapy. **h** Photographs of tumors removed from the mice in each group (*n* = 5). **i** The statistical plot of the tumor weights of the four groups (*n* = 5). **j** Changes in mouse body weight (*n* = 5). **k**, **l** Flow cytometry of CD8 + T cells of spleens and the statistical analysis of the results (*n* = 5). **m** The model shows the regulation of PD-L1 stability by USP8 in pancreatic cancer. USP8 inhibitor treatment downregulates PD-L1 protein levels via degradation, leading to activation of the cytotoxic T-cells. The results are displayed as the means ± SD from representative experiments in (**e**, **f**, **i, j** and **l**). The data represent three independent experiments. *p < 0.05, **p < 0.01, ***p < 0.001, ****p < 0.0001 assessed via a two-tailed *t* test; ns not significant.

To further determine the role of PD-L1 in the effect of the combination therapy comprising the USP8 inhibitor and αPD-L1, we constructed a *Cd274* knockout (KO) KPC cell line using CRISPR/Cas9 KO plasmid, which was verified by western blotting analysis and flow cytometry (Fig. 8a, b). We constructed orthotopic parental and *Cd274* KO KPC cell-bearing mice, which were administrated with the combination therapy (Fig. 8c). We observed no significant reduction in tumor size and weight in the *Cd274* KO group using the combination therapy compared with that in the parental group (Fig. 8d, e). Additionally, at the study endpoint, the average weight of the mice was not significantly different among the groups (Fig. 8f). Additionally, in vitro, the USP8 inhibitor-αPD-L1 combination therapy did not significantly improve the killing of tumor cells by activated T cells in *Cd274* KO KPC cells compared to parental KPC cells (Fig. S14a, b). Moreover, to further confirm that CD8 + T cells were the mediators of anti-tumor immunity, we depleted CD8 + T cells prior to inoculation with KPC cells and combined USP8 inhibitor and αPD-L1 treatment (Fig. 8g). The difference in tumor burden between the ctrl group and the combination therapy group was absent after treatment of the KPC tumor mice with CD8-depleting antibodies (Fig. 8h, i). The treated animals showed no weight loss (Fig. 8j). The depletion of CD8 + T cells in the spleens of the mice was confirmed by flow cytometry (Fig. 8k, l). Overall, the above results verified that the anti-tumor immunity caused by the combined therapy depends on the PD-L1 pathway and CD8 + T cells.

Collectively, USP8 stabilized PD-L1 by inhibiting its degradation via the ubiquitin-proteasome pathway in pancreatic cancer cells. Combination therapy comprising a USP8 inhibitor and αPD-L1 suppressed pancreatic tumor growth and activated cytotoxic T-lymphocytes by regulating PD-L1 stability (Fig. 8m).

## Discussion

Deubiquitylating enzymes (DUBs) can remove the ubiquitin moieties from substrates to alter the state of the protein and consequently maintain a dynamic balance of intracellular protein quantity and activity [27–29]. A study showed that mice with specific deletion of USP8 + T cells exhibited dysfunctional Tregs and inflammatory bowel disease [30], which suggested that USP8 might be associated with T cell function. Several targets, including HER-2, TRAF6, and Akt, are implicated in USP8-mediated tumorigenesis [31–33]. USP8 inhibited the migration and proliferation of HER-2 positive gastric cancer cells through the PI3K/AKT signaling pathway [31]. Meanwhile, USP8 regulated liver cancer cell progression via inhibition of TRAF6-mediated signaling [32]. Additionally, USP8 linked the PTEN-Akt-AIP4 pathway to the regulation of FLIPS stability and TRAIL sensitivity in Glioblastoma Multiforme [33]. Herein, the results showed that USP8 levels were upregulated in clinical pancreatic tumor samples compared with those in normal tissues, meanwhile, we assessed USP8 expression in different tumor molecular and immune subtypes, attributed to the complicated subtypes in PDAC [34]. We noted a correlation between high USP8 expression and poor TNM stage in patients with pancreatic cancer. Moreover, we found that USP8 deficiency inhibited pancreatic tumor proliferation and extended overall survival by improving anti-tumor immunogenicity.

The most crucial issue in anti-tumor treatment is fixing the therapeutic targets, and increasing studies report that immune checkpoint blockade (ICB) can stimulate the immune system using PD-L1 antibodies, while tumor cells often resist anti-PD-L1 therapy via immune evasion. Hence, discovering new biomarkers that can reflect PD-L1 expression levels and the density of tumor-infiltrating lymphocytes (TILs) might effectively predict the effectiveness of blocking PD-1/PD-L1 and the correlation with anti-PD-L1 therapy [35]. In this study, we demonstrated that in pancreatic tumor cell lines, USP8 deficiency induced a time and dose-dependent decrease in the PD-L1 protein level and increased the amount and function of tumor-infiltrated activated T-cells. Meanwhile, USP8 intervention might increase the efficacy of PD-L1 blockade. Therefore, USP8 could represent a therapeutic target and potential biomarker in pancreatic tumors. Membrane protein biogenesis is involved in multiple and consecutive steps, including in the plasma membrane, ER, and Golgi [36]. Hence, proteins can be degraded at each step to regulate their abundance [37]. However, the specific pathway that regulates PD-L1 ubiquitination, in which USP8 inhibits PD-L1 degradation, remains unclear. Determining this pathway will lead to a deeper understanding of the mechanism by which PD-L1 is enriched in tumor cell membranes. Furthermore, A study showed that epidermal growth factor (EGF) can stabilize PD-L1 expression and an EGF inhibitor (gefitinib) might enhance anti-tumor immunity in syngeneic mouse models [38]. However, we demonstrated that in pancreatic cancer, *USP8* knockdown and USP8 inhibition did not affect EGFR expression levels. Moreover, USP8 inhibitors can be expected to be combined with biomaterials in the future to develop drugs with better targeting and fewer toxic side effects.

In conclusion, we revealed that USP8 is a novel PD-L1 deubiquitinating enzyme that upregulates PD-L1 levels by inhibiting the ubiquitination-regulated proteasome degradation pathway, thereby promoting pancreatic tumor growth via immune evasion. Combination therapy with a USP8 inhibitor and αPD-L1 downregulated PD-L1 protein levels, leading to activation of cytotoxic T-cells. Collectively, targeting USP8 in combination with other anti-tumor drugs might be a potential strategy in cancer immunotherapy to improve patient outcomes.

## Supplementary information

Supplementary Figure 1

Supplementary Figure 2

Supplementary Figure 3

Supplementary Figure 4

Supplementary Figure 5

Supplementary Figure 6

Supplementary Figure 7

Supplementary Figure 8

Supplementary Figure 9

Supplementary Figure 10

Supplementary Figure 11

Supplementary Figure 12

Supplementary Figure 13

Supplementary Figure 14

Supplementary Figure 15

Supplementary Table

Supplementary Information

Reproducibility Checklist

Original Data File

## Data Availability

The data are available from the corresponding author on reasonable request.

## References

[1] McGuigan A, Kelly P, Turkington RC, Jones C, Coleman HG, McCain RS (2018). Pancreatic cancer: a review of clinical diagnosis, epidemiology, treatment and outcomes. World J Gastroenterol.

[2] Chen L, Han X (2015). Anti-PD-1/PD-L1 therapy of human cancer: past, present, and future. J Clin Invest.

[3] Constantinidou A, Alifieris C, Trafalis DT (2019). Targeting Programmed Cell Death -1 (PD-1) and Ligand (PD-L1): a new era in cancer active immunotherapy. Pharm Ther.

[4] Vranic S, Cyprian FS, Gatalica Z, Palazzo J (2021). PD-L1 status in breast cancer: current view and perspectives. Semin Cancer Biol.

[5] Ohaegbulam KC, Assal A, Lazar-Molnar E, Yao Y, Zang X (2015). Human cancer immunotherapy with antibodies to the PD-1 and PD-L1 pathway. Trends Mol Med.

[6] Huber M, Brehm CU, Gress TM, Buchholz M, Alashkar Alhamwe B, von Strandmann EP (2020). The Immune Microenvironment in Pancreatic Cancer. Int J Mol Sci.

[7] Zhang J, Dang F, Ren J, Wei W (2018). Biochemical aspects of PD-L1 regulation in cancer immunotherapy. Trends Biochem Sci.

[8] Hsu J-M, Li C-W, Lai Y-J, Hung M-C (2018). Posttranslational modifications of PD-L1 and their applications in cancer therapy. Cancer Res.

[9] Li S-M, Zhou J, Wang Y, Nie R-C, Chen J-W, Xie D (2020). Recent findings in the posttranslational modifications of PD-L1. J Oncol.

[10] Hu X, Wang J, Chu M, Liu Y, Wang Z-W, Zhu X (2021). Emerging role of ubiquitination in the regulation of PD-1/PD-L1 in cancer immunotherapy. Mol Ther.

[11] Shackleford TJ, Claret FX (2010). JAB1/CSN5: a new player in cell cycle control and cancer. Cell Div.

[12] Zhang X, Huang X, Xu J, Li E, Lao M, Tang T (2021). NEK2 inhibition triggers anti-pancreatic cancer immunity by targeting PD-L1. Nat Commun.

[13] Huang X, Zhang Q, Lou Y, Wang J, Zhao X, Wang L (2019). USP22 deubiquitinates CD274 to suppress anticancer immunity. Cancer Immunol Res.

[14] Wilkinson KD (1997). Regulation of ubiquitin-dependent processes by deubiquitinating enzymes. FASEB J.

[15] Islam MDT, Chen F, Chen H (2021). The oncogenic role of ubiquitin specific peptidase (USP8) and its signaling pathways targeting for cancer therapeutics. Arch Biochem Biophysics.

[16] Naviglio S, Mattecucci C, Matoskova B, Nagase T, Nomura N, Di Fiore PP (1998). UBPY: a growth-regulated human ubiquitin isopeptidase. EMBO J.

[17] Zhou M, Chen F, Chen H (2014). Ubiquitination involved enzymes and cancer. Med Oncol.

[18] Bland T, Sahin GS, Zhu M, Dillon C, Impey S, Appleyard SM (2019). USP8 deubiquitinates the leptin receptor and is necessary for leptin-mediated synapse formation. Endocrinology.

[19] Rong Z, Zhu Z, Cai S, Zhang B (2020). Knockdown of USP8 inhibits the growth of lung cancer cells. Cancer Manag Res.

[20] Sun J, Shen D, Gao Y, Zheng Y, Zhao L, Maa M (2020). Downregulation of USP8 suppresses HER-3 positive gastric cancer cells proliferation. Onco Targets Ther.

[21] Shin S, Kim K, Kim H-R, Ylaya K, Do S-I, Hewitt SM (2020). Deubiquitylation and stabilization of Notch1 intracellular domain by ubiquitin-specific protease 8 enhance tumorigenesis in breast cancer. Cell Death Differ.

[22] Qiu H, Kong J, Cheng Y, Li G (2018). The expression of ubiquitin-specific peptidase 8 and its prognostic role in patients with breast cancer. J Cell Biochem.

[23] Zhang Q, Lou Y, Zhang J, Fu Q, Wei T, Sun X (2017). Hypoxia-inducible factor-2α promotes tumor progression and has crosstalk with Wnt/β-catenin signaling in pancreatic cancer. Mol Cancer.

[24] Tiwari A, Tashiro K, Dixit A, Soni A, Vogel K, Hall B (2020). Loss of HIF1A from pancreatic cancer cells increases expression of PPP1R1B and degradation of p53 to promote invasion and metastasis. Gastroenterology.

[25] Lamberto I, Liu X, Seo H-S, Schauer NJ, Iacob RE, Hu W (2017). Structure-guided development of a potent and selective non-covalent active-site inhibitor of USP7. Cell Chem Biol.

[26] Pfaffl MW (2001). A new mathematical model for relative quantification in real-time RT-PCR. Nucleic Acids Res.

[27] Ventii KH, Wilkinson KD (2008). Protein partners of deubiquitinating enzymes. Biochem J.

[28] Haq S, Suresh B, Ramakrishna S (2018). Deubiquitylating enzymes as cancer stem cell therapeutics. Biochim Biophys Acta Rev Cancer.

[29] Nijman SMB, Luna-Vargas MPA, Velds A, Brummelkamp TR, Dirac AMG, Sixma TK (2005). A genomic and functional inventory of deubiquitinating enzymes. Cell.

[30] Dufner A, Kisser A, Niendorf S, Basters A, Reissig S, Schönle A (2015). The ubiquitin-specific protease USP8 is critical for the development and homeostasis of T cells. Nat Immunol.

[31] Sun J, Shen D, Zheng Y, Ren H, Liu H, Chen X (2020). USP8 inhibitor duppresses HER-2 positive gastric cancer cell proliferation and metastasis via the PI3K/AKT signaling pathway. Onco Targets Ther.

[32] Kim M-J, Choi B, Kim JY, Min Y, Kwon DH, Son J (2022). USP8 regulates liver cancer progression via the inhibition of TRAF6-mediated signal for NF-κB activation and autophagy induction by TLR4. Transl Oncol.

[33] Panner A, Crane CA, Weng C, Feletti A, Fang S, Parsa AT (2010). Ubiquitin-specific protease 8 links the PTEN-Akt-AIP4 pathway to the control of FLIPS stability and TRAIL sensitivity in glioblastoma multiforme. Cancer Res.

[34] Collisson EA, Bailey P, Chang DK, Biankin AV (2019). Molecular subtypes of pancreatic cancer. Nat Rev Gastroenterol Hepatol.

[35] Yi M, Jiao D, Xu H, Liu Q, Zhao W, Han X (2018). Biomarkers for predicting efficacy of PD-1/PD-L1 inhibitors. Mol Cancer.

[36] Guna A, Hegde RS (2018). Transmembrane domain recognition during membrane protein biogenesis and quality control. Curr Biol.

[37] Avci D, Lemberg MK (2015). Clipping or extracting: two ways to membrane protein degradation. Trends Cell Biol.

[38] Li C-W, Lim S-O, Xia W, Lee H-H, Chan L-C, Kuo C-W (2016). Glycosylation and stabilization of programmed death ligand-1 suppresses T-cell activity. Nat Commun.

