# Supplementary figures and images for: Targeting ubiquitin-specific protease 8 sensitizes anti-programmed death-ligand 1 immunotherapy of pancreatic cancer
Source: Cell Death Differ. 2022 Dec 20;30(2):560–75. doi: 10.1038/s41418-022-01102-z (PMC9950432; doi:10.1038/s41418-022-01102-z)

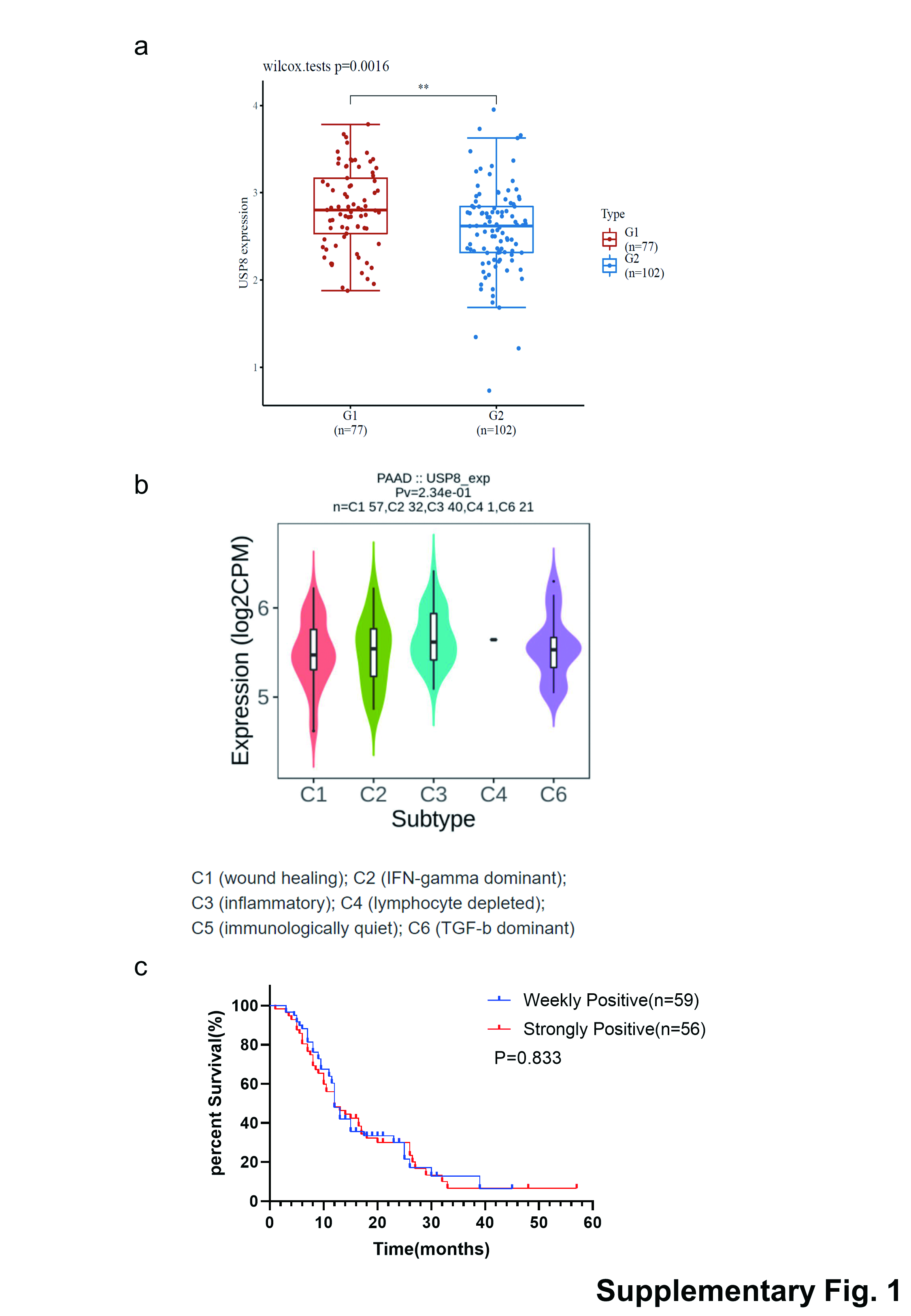

Supplement: Supplementary file 1 — Supplementary Figure 1 [file 41418_2022_1102_MOESM1_ESM.tif]

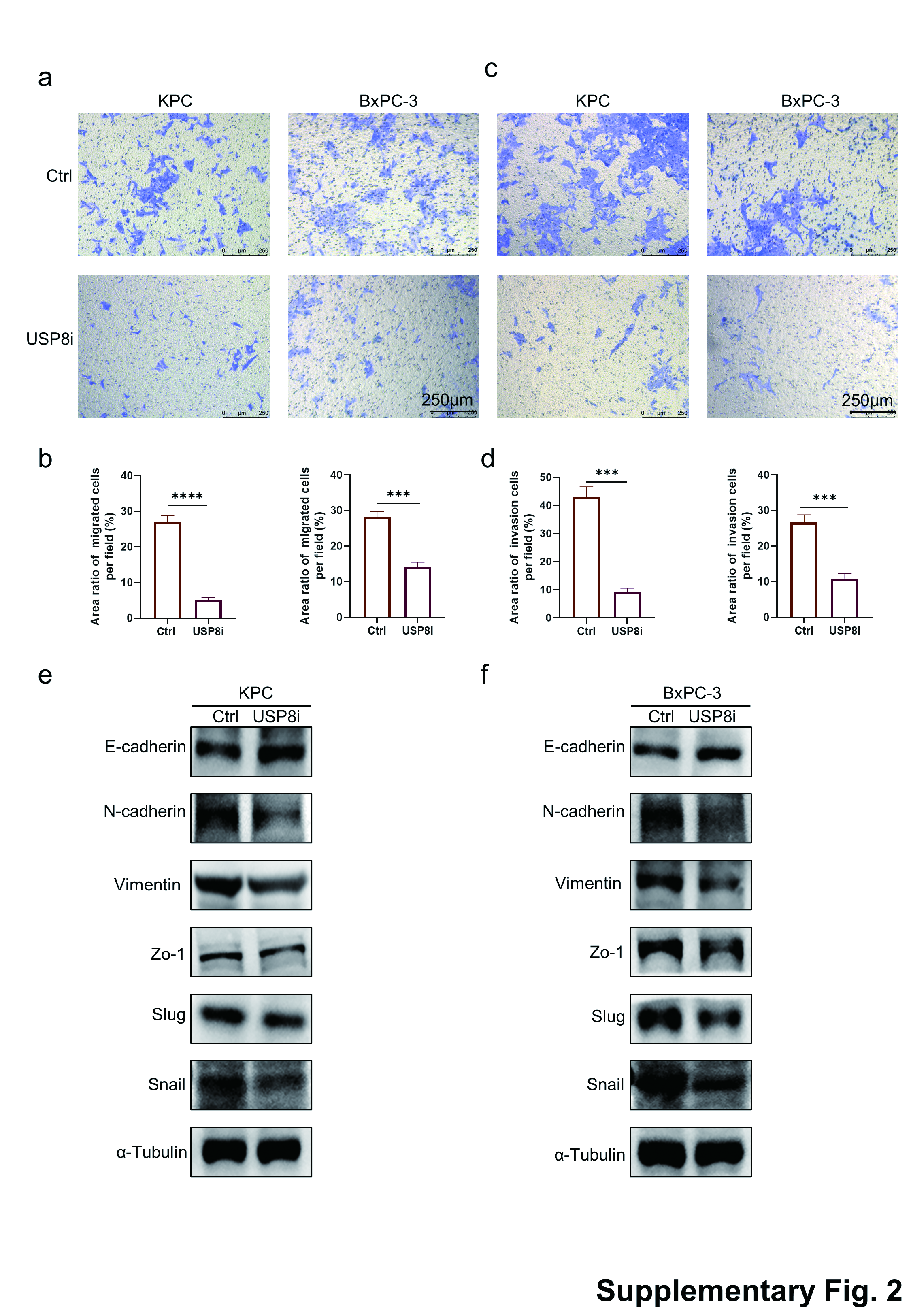

Supplement: Supplementary file 2 — Supplementary Figure 2 [file 41418_2022_1102_MOESM2_ESM.tif]

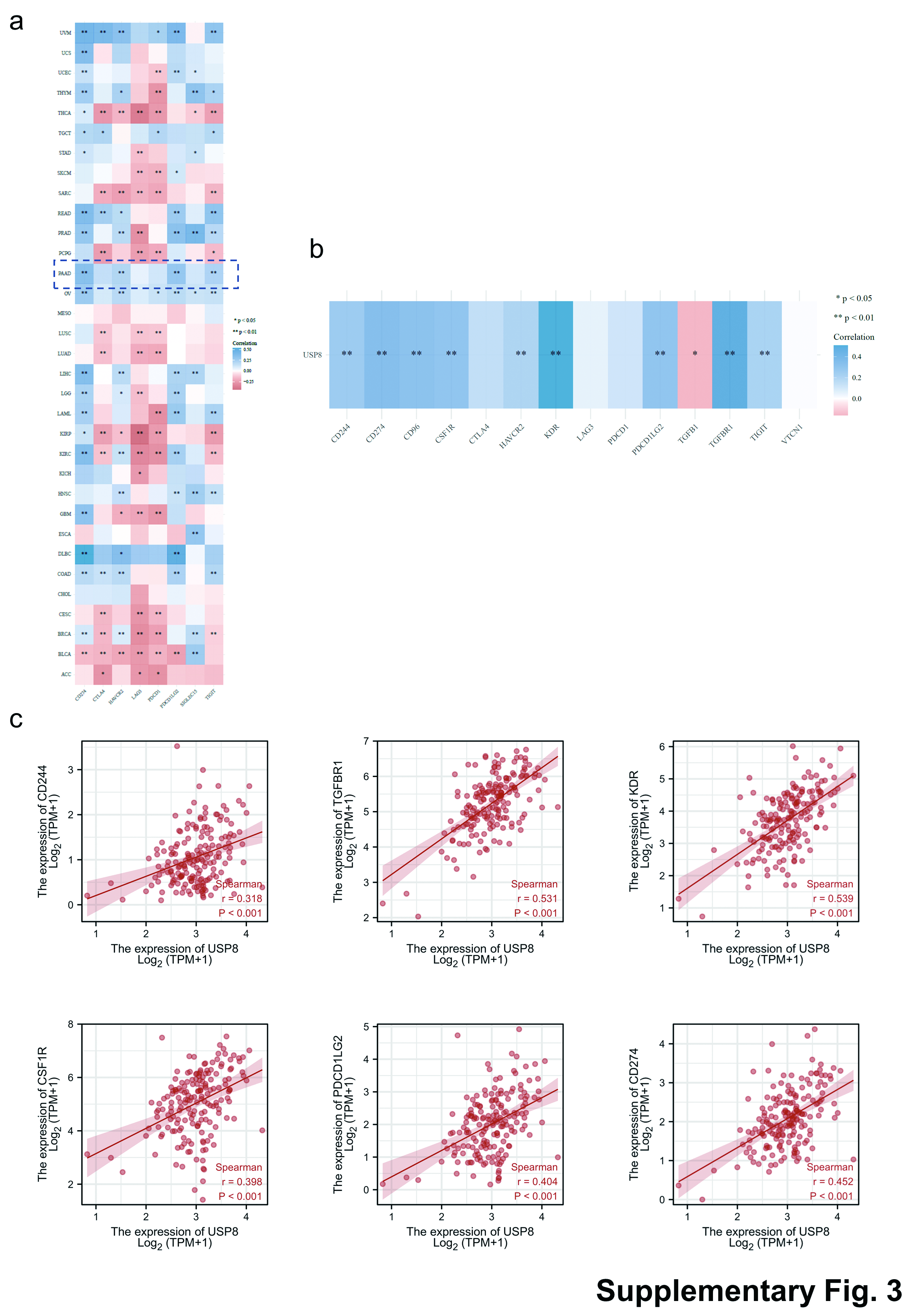

Supplement: Supplementary file 3 — Supplementary Figure 3 [file 41418_2022_1102_MOESM3_ESM.tif]

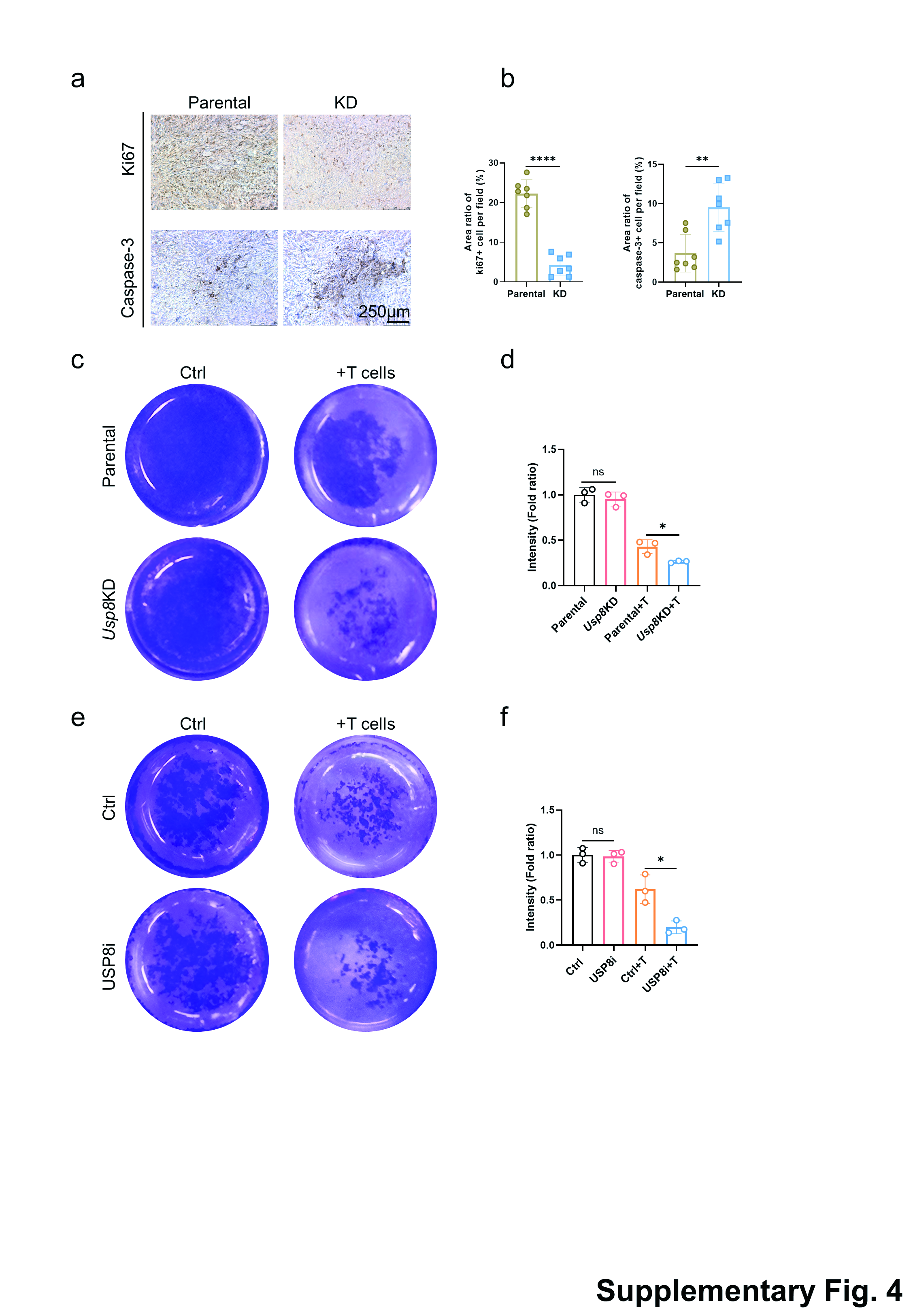

Supplement: Supplementary file 4 — Supplementary Figure 4 [file 41418_2022_1102_MOESM4_ESM.tif]

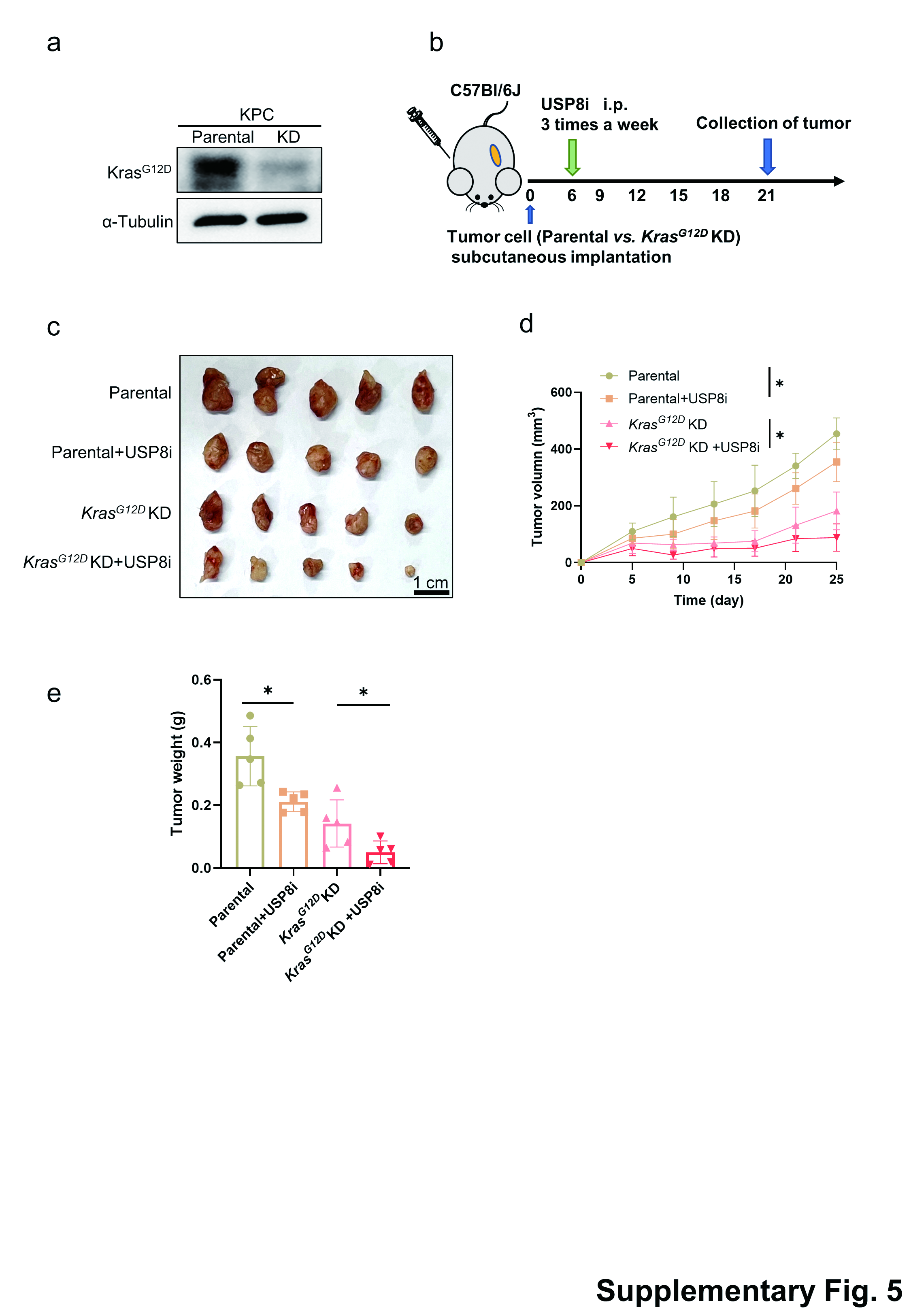

Supplement: Supplementary file 5 — Supplementary Figure 5 [file 41418_2022_1102_MOESM5_ESM.tif]

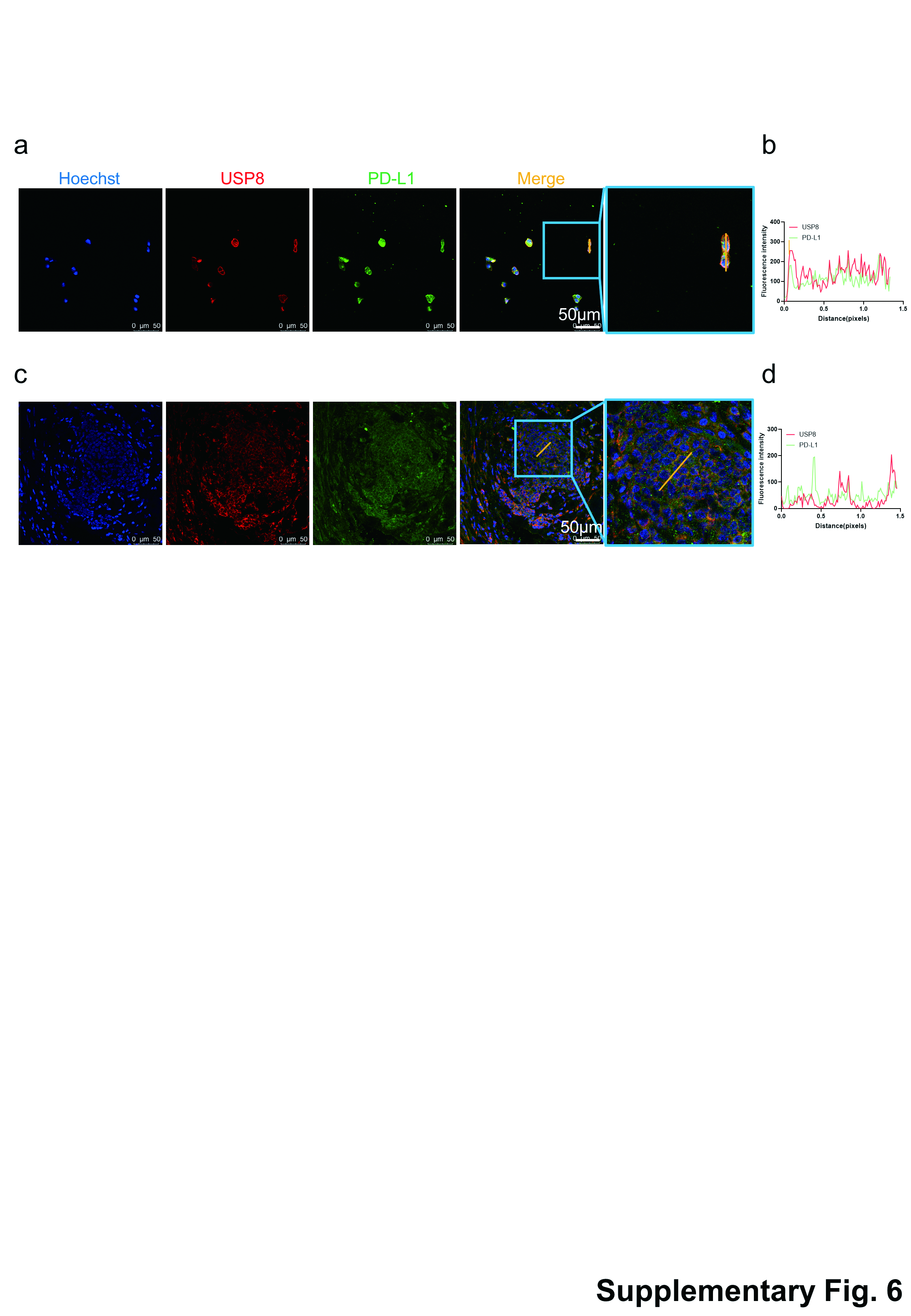

Supplement: Supplementary file 6 — Supplementary Figure 6 [file 41418_2022_1102_MOESM6_ESM.tif]

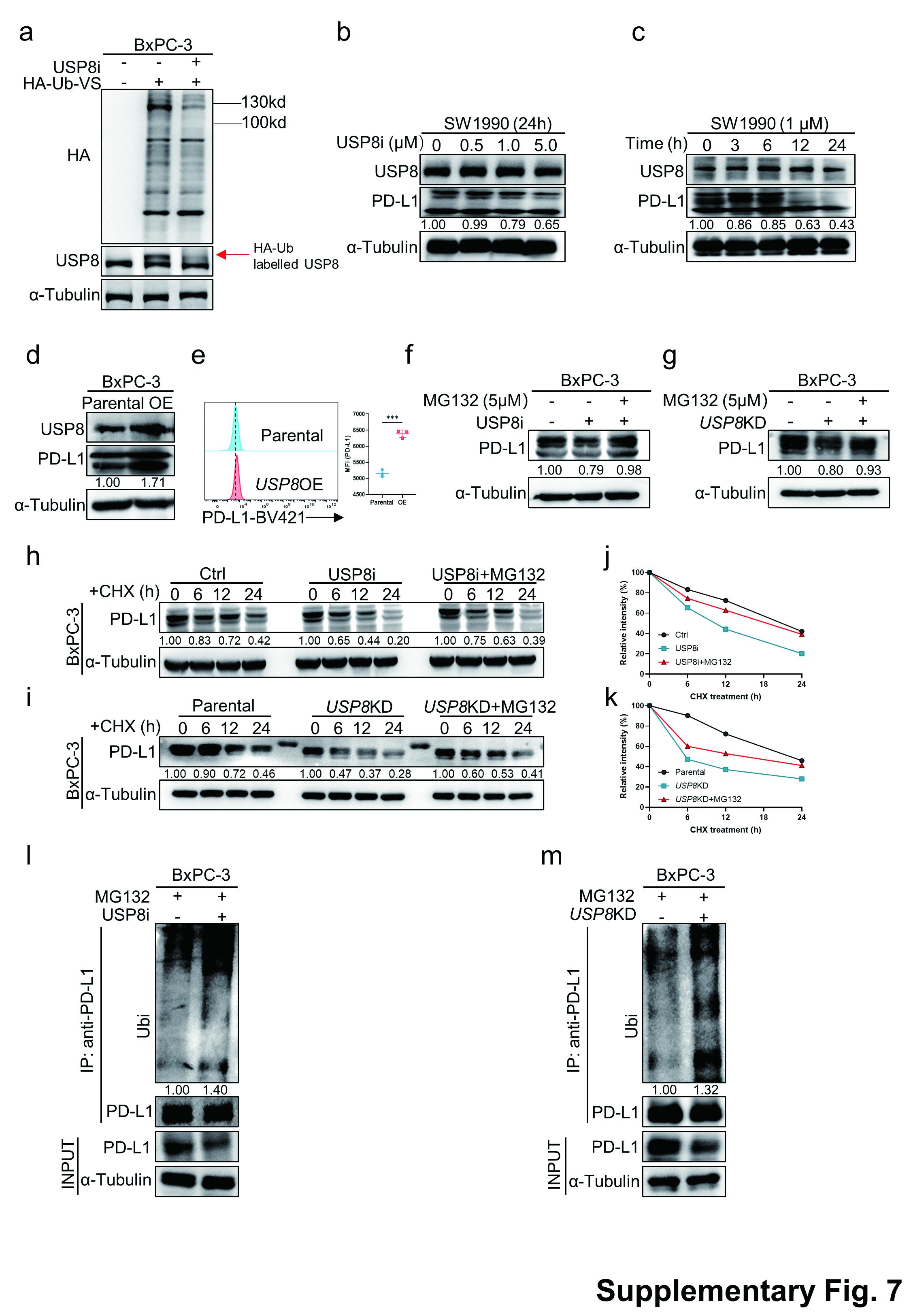

Supplement: Supplementary file 7 — Supplementary Figure 7 [file 41418_2022_1102_MOESM7_ESM.tif]

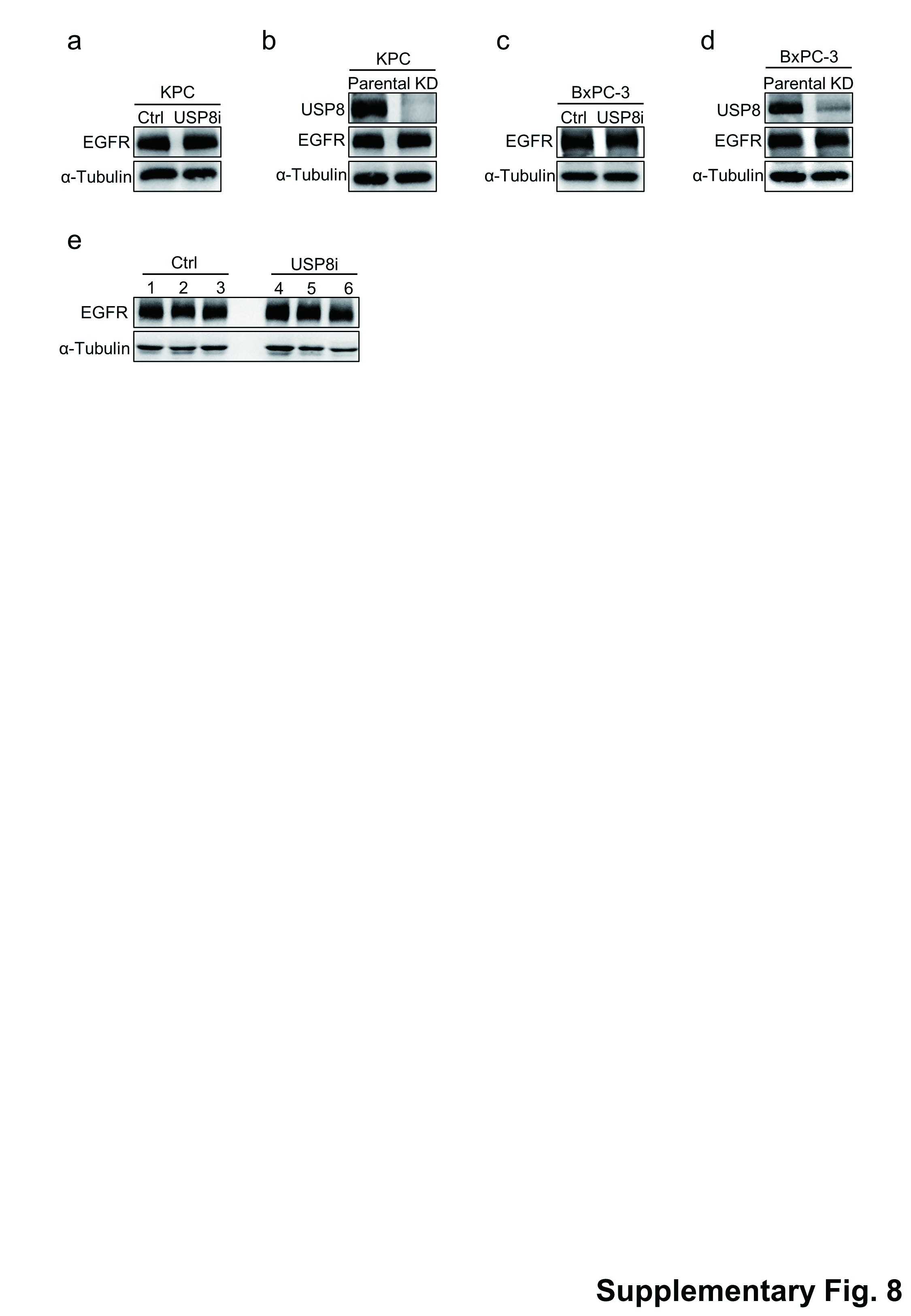

Supplement: Supplementary file 8 — Supplementary Figure 8 [file 41418_2022_1102_MOESM8_ESM.tif]

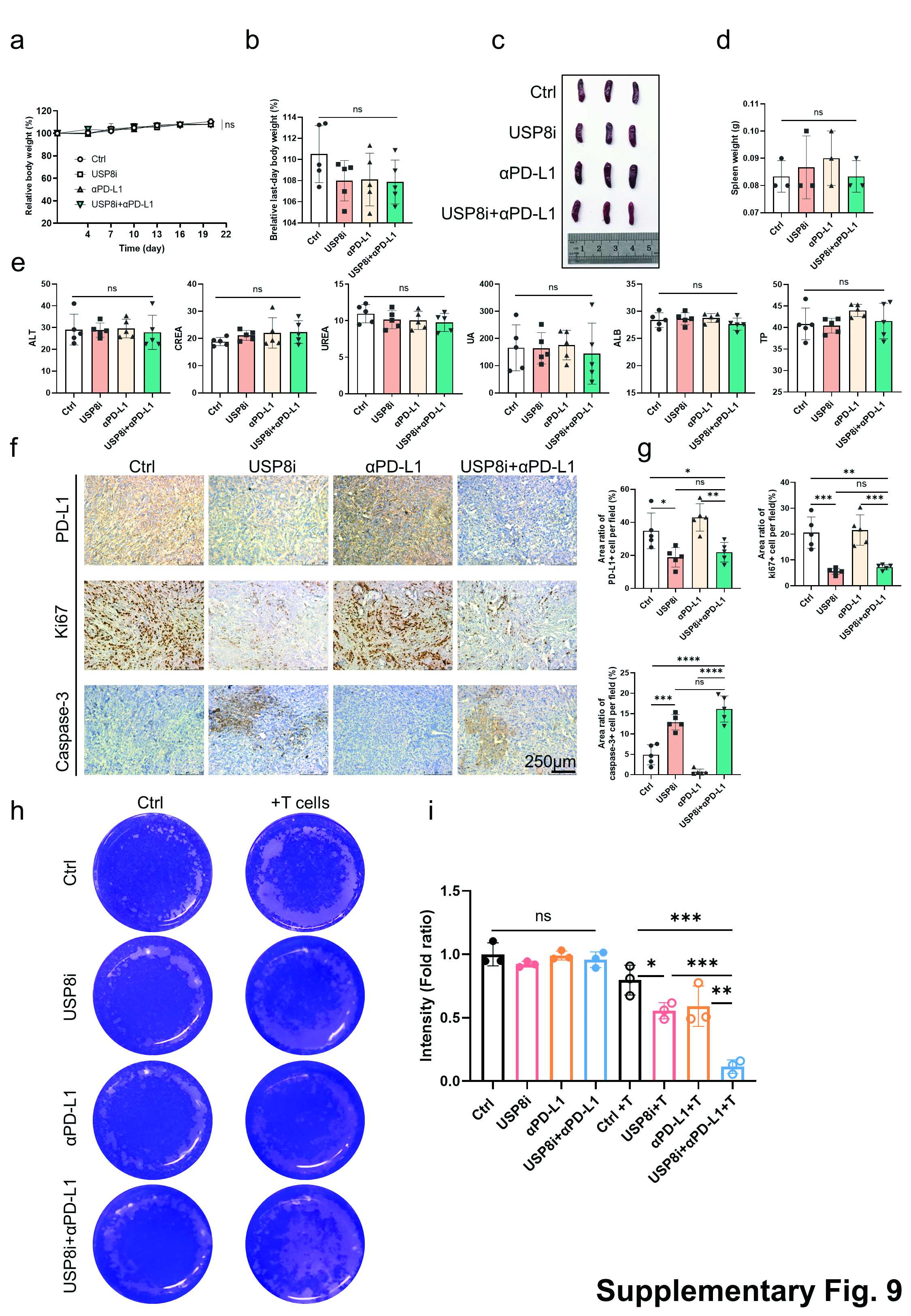

Supplement: Supplementary file 9 — Supplementary Figure 9 [file 41418_2022_1102_MOESM9_ESM.tif]

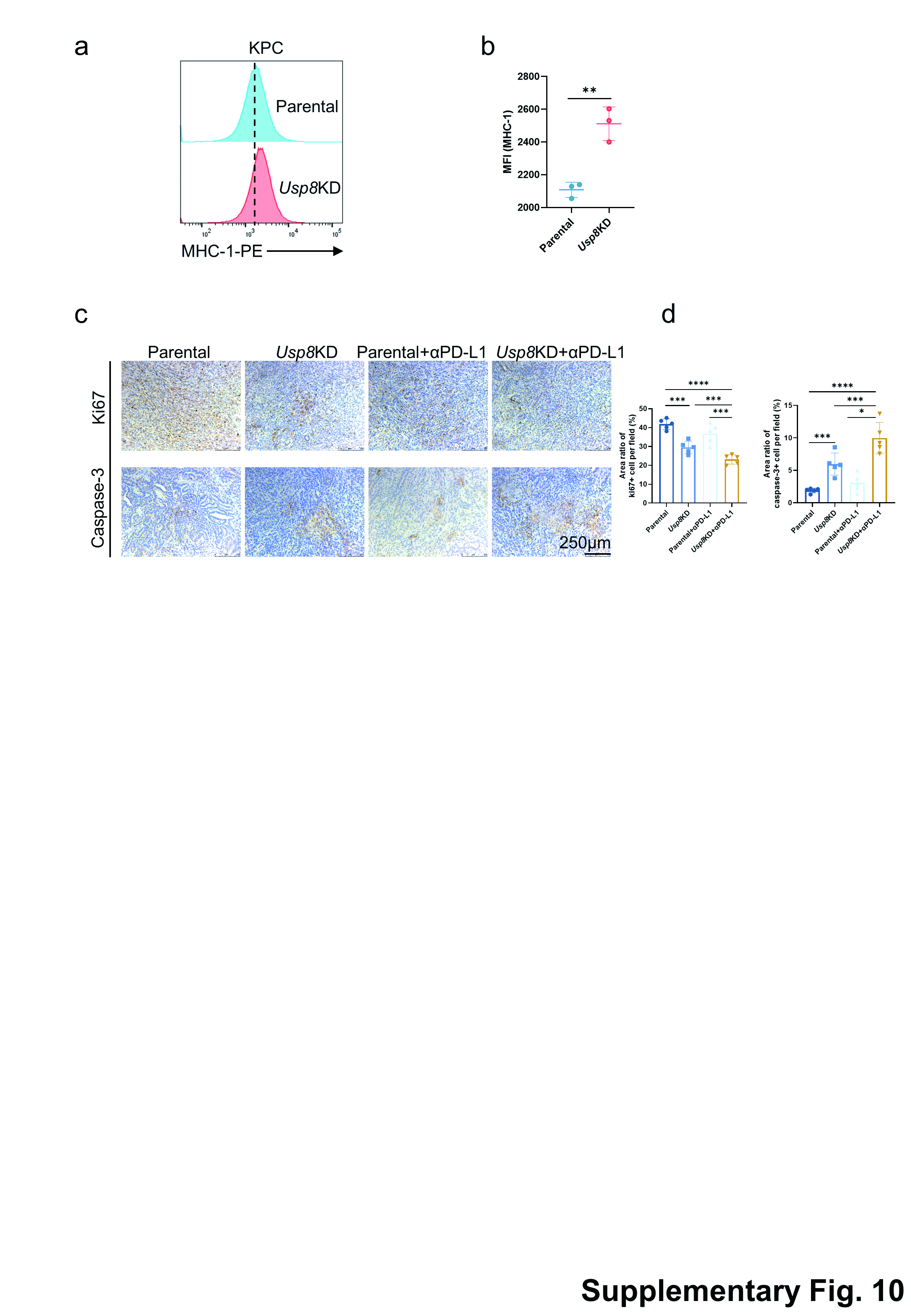

Supplement: Supplementary file 10 — Supplementary Figure 10 [file 41418_2022_1102_MOESM10_ESM.tif]

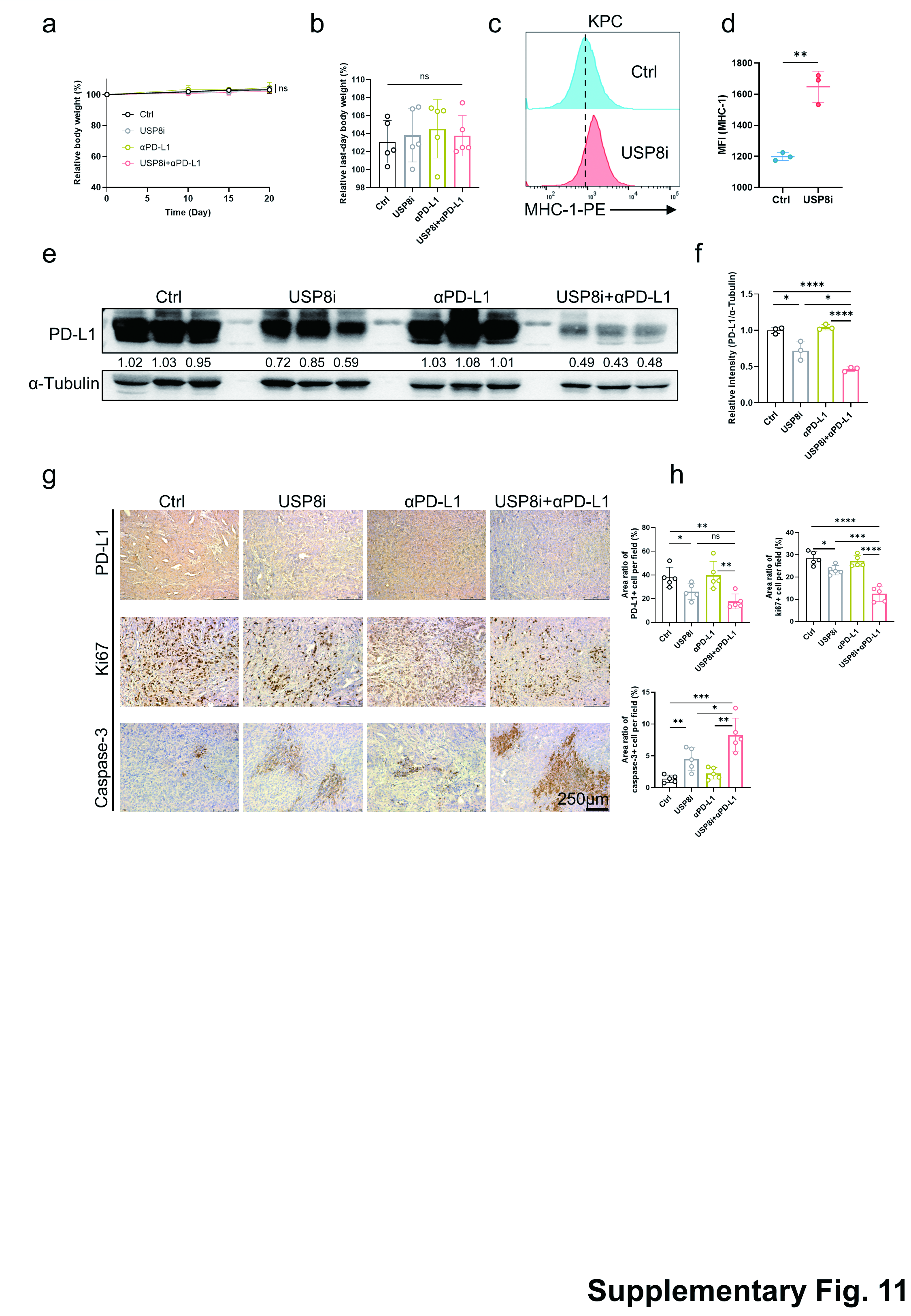

Supplement: Supplementary file 11 — Supplementary Figure 11 [file 41418_2022_1102_MOESM11_ESM.tif]

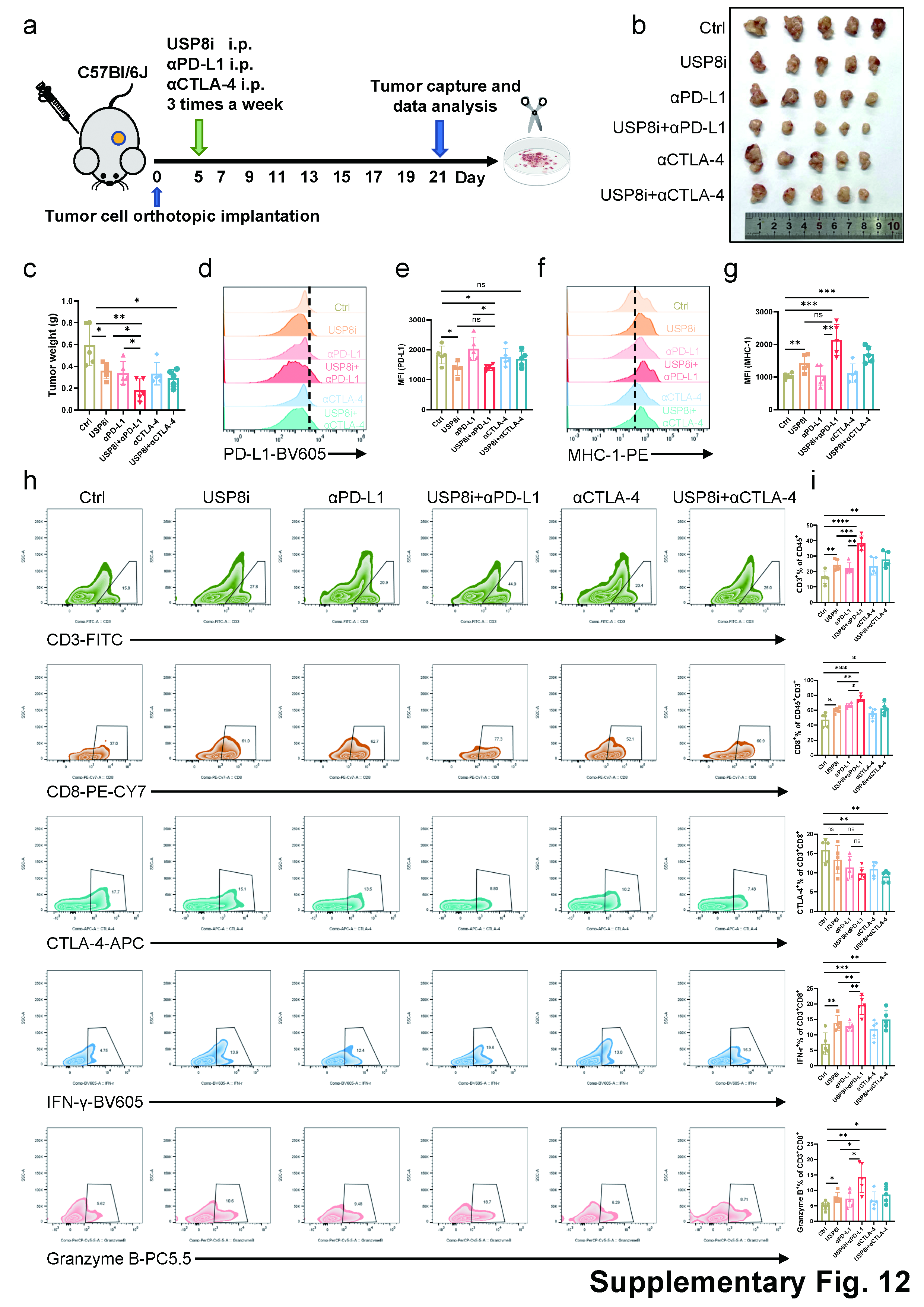

Supplement: Supplementary file 12 — Supplementary Figure 12 [file 41418_2022_1102_MOESM12_ESM.tif]

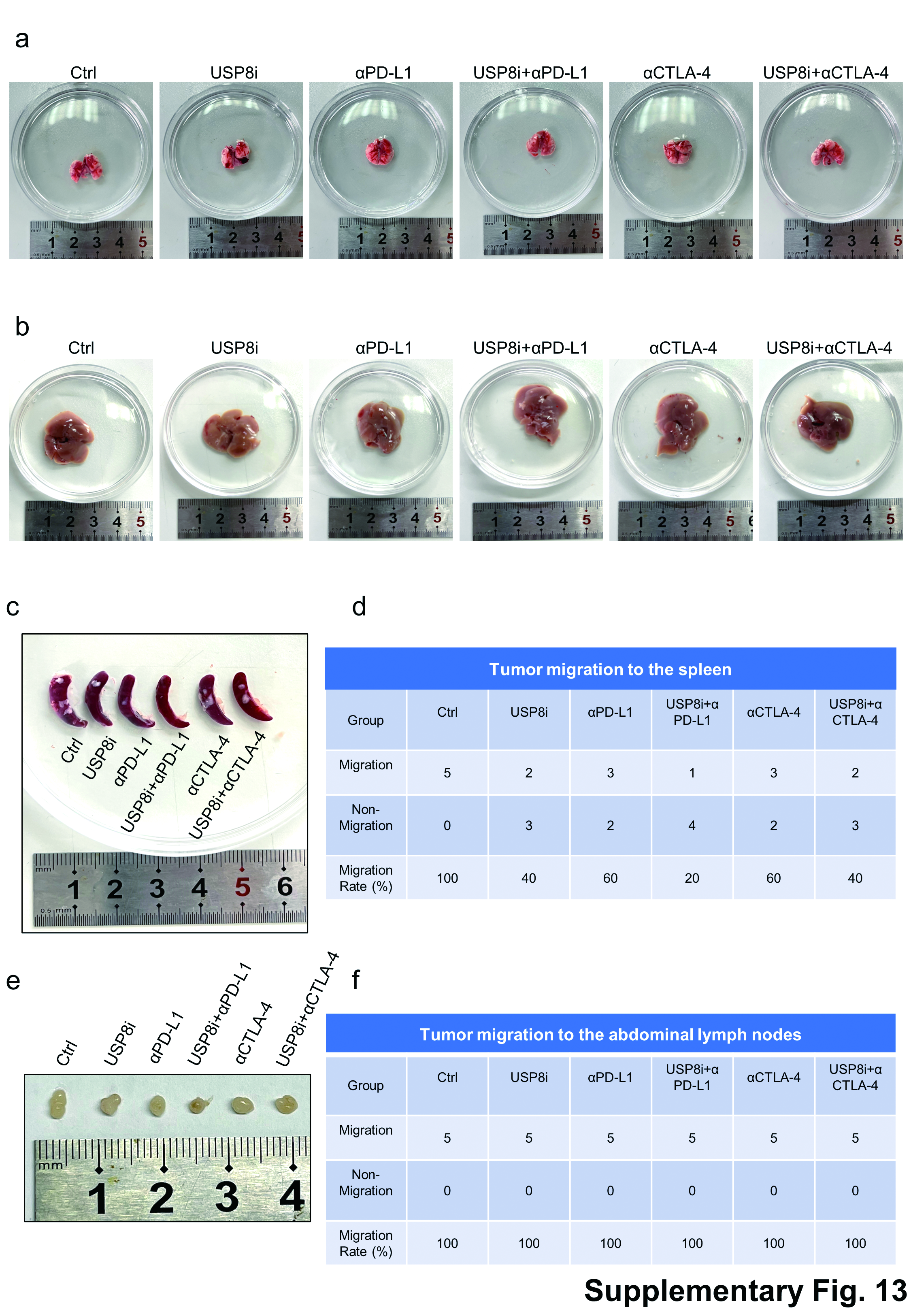

Supplement: Supplementary file 13 — Supplementary Figure 13 [file 41418_2022_1102_MOESM13_ESM.tif]

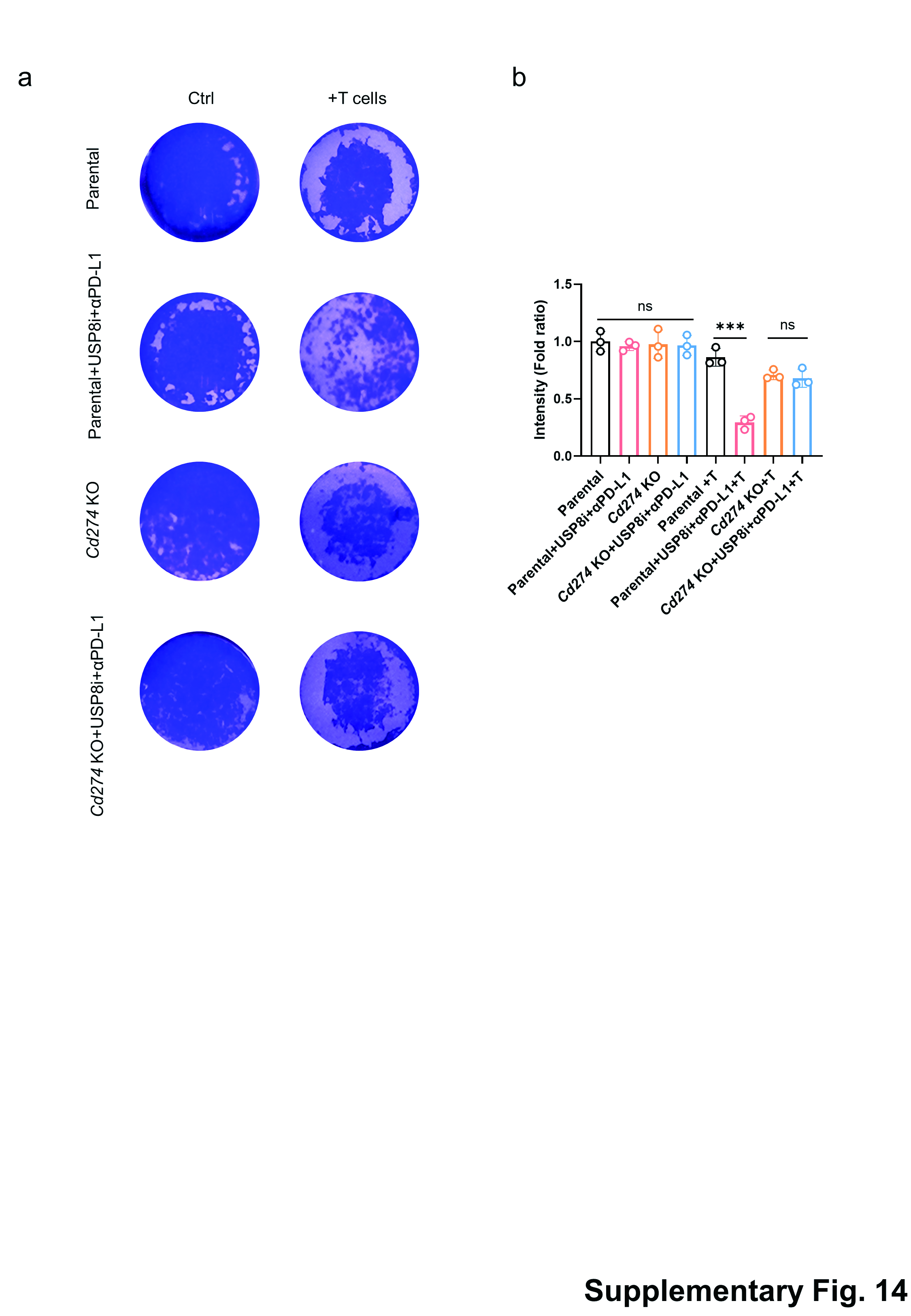

Supplement: Supplementary file 14 — Supplementary Figure 14 [file 41418_2022_1102_MOESM14_ESM.tif]

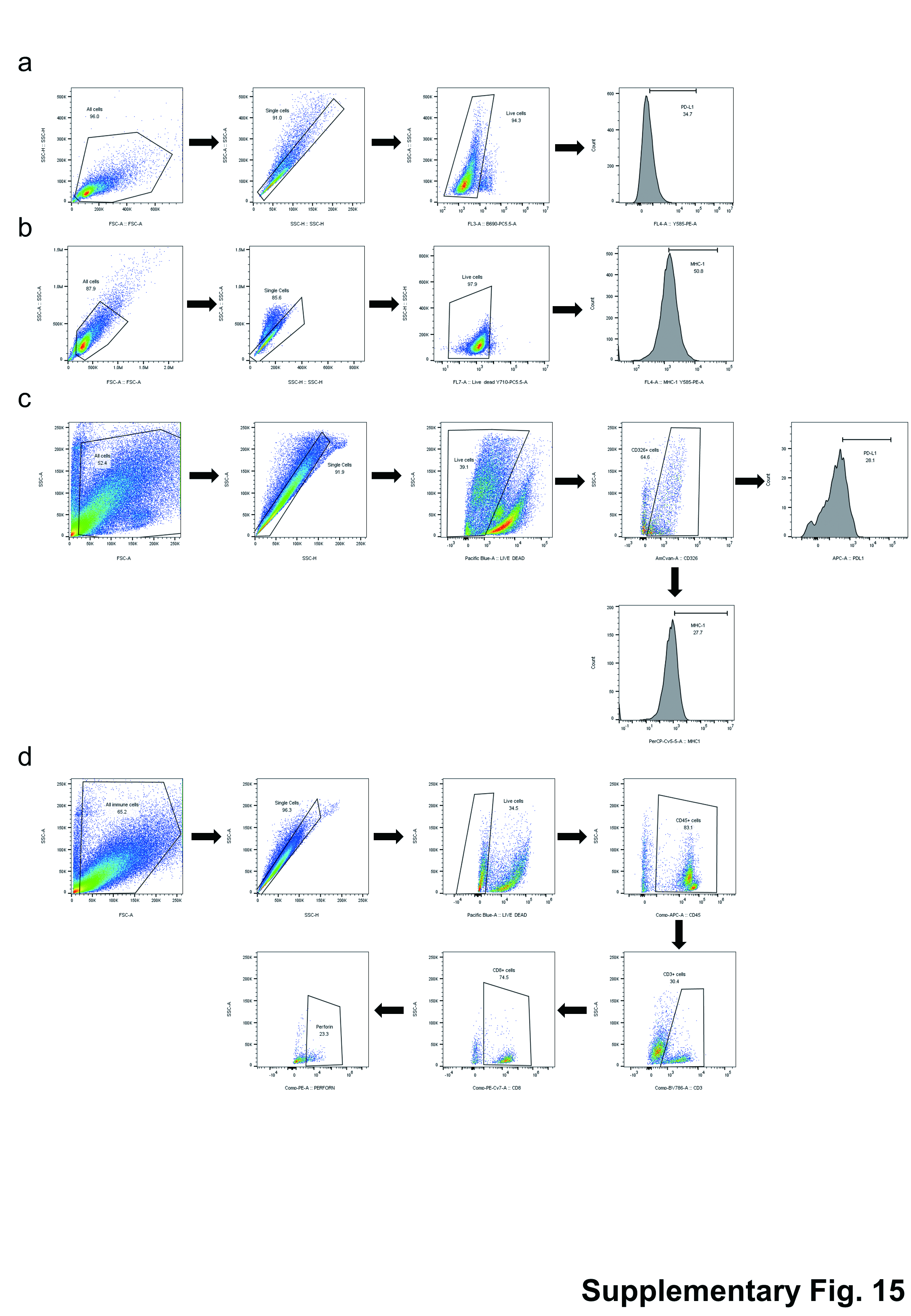

Supplement: Supplementary file 15 — Supplementary Figure 15 [file 41418_2022_1102_MOESM15_ESM.tif]
