# Supplementary material for: Targeting ubiquitin-specific protease 8 sensitizes anti-programmed death-ligand 1 immunotherapy of pancreatic cancer
Source: Cell Death Differ. 2022 Dec 20;30(2):560–75. doi: 10.1038/s41418-022-01102-z (PMC9950432; doi:10.1038/s41418-022-01102-z)
Supplement: Supplementary file 16 — Supplementary Table [file 41418_2022_1102_MOESM16_ESM.docx]

| **Supplementary Table 1 Clinicopathological relevance of USP8 in patients with PDAC.** | | | |
| --- | --- | --- | --- |
| **Variable** | **USP8** | | |
|  | **Low (H-score ≤ 90) High (H-score > 90) P value** | | |
| *Sex* |  |  | 0.676 |
| Male, n (%) | 43 (55.8%) | 45 (59.2%) |  |
| Female, n (%) | 34 (44.2%) | 31 (40.8%) |  |
| *Age, years* |  |  | 0.975 |
| > 60 | 65 (86.7%) | 66 (86.8%) |  |
| ≤ 60 | 10 (13.3%) | 10 (13.2%) |  |
| *BMI, kg/m^2^* |  |  | 0.263 |
| < 18.5 | 10 (14.5%) | 8 (11.9%) |  |
| 18.5-23.9 | 42 (60.9%) | 36 (53.7%) |  |
| > 23.9 | 17 (24.6%) | 23 (34.3%) |  |
| *TNM stage* |  |  | **0.041** |
| Ⅰ | 17 (22.4%) | 8 (10.5%) |  |
| Ⅱ | 42 (55.3%) | 48 (63.2%) |  |
| Ⅲ | 16 (21.1%) | 12 (15.8%) |  |
| Ⅳ | 1 (1.3%) | 8 (10.5%) |  |
| *Vascular invasion* |  |  | 0.812 |
| Yes | 36 (48.0%) | 35 (46.1%) |  |
| No | 36 (48.0%) | 35 (46.1%) |  |
| *Nerve invasion* |  |  | 0.88 |
| Yes | 57 (76.0%) | 48 (63.2%) |  |
| No | 18 (24.0%) | 28 (36.8%) |  |
| *Lymphatic metastasis* |  |  | 0.745 |
| Yes | 44 (57.9%) | 42 (55.3%) |  |
| No | 32 (42.1%) | 34 (44.7%) |  |
| *Serum CA19-9, U/ml* |  |  | 0.103 |
| ≥ 37 | 65 (86.7%) | 58 (76.3%) |  |
| < 37 | 10 (13.3%) | 18 (23.7%) |  |
| *Serum CA12-5, U/ml* |  |  | 0.688 |
| ≥ 35 | 18 (27.7%) | 18 (24.7%) |  |
| < 35 | 47 (72.3%) | 55 (75.3%) |  |
| *Serum CEA, U/ml* |  |  | 0.486 |
| ≥ 5 | 21 (31.3%) | 27 (37.0%) |  |
| < 5 | 46 (68.7%) | 46 (63.0%) |  |
| *Tumor differentiation* |  |  | 0.685 |
| Well | 4 (5.4%) | 4 (5.5%) |  |
| Moderate | 45 (60.8%) | 47 (64.4%) |  |
| Poor | 25 (33.8%) | 22 (30.1%) |  |
| *Recurrence* |  |  | 0.878 |
| Yes | 46 (76.7%) | 43 (75.4%) |  |
| No | 14 (23.3%) | 14 (24.6%) |  |

One-way and repeated-measures analysis of variance (ANOVA) was performed in Table 1. The bold values indicate p < 0.05.
