# Supplementary material for: Targeting ubiquitin-specific protease 8 sensitizes anti-programmed death-ligand 1 immunotherapy of pancreatic cancer
Source: Cell Death Differ. 2022 Dec 20;30(2):560–75. doi: 10.1038/s41418-022-01102-z (PMC9950432; doi:10.1038/s41418-022-01102-z)
Supplement: Supplementary file 17 — Supplementary Information [file 41418_2022_1102_MOESM17_ESM.docx]

**Supplementary Information**

**Supplementary figure legends**

**Supplementary Fig.1** The connection of USP8 expression with the immunogenic subtype and overall survival. **a**, **b** Bioinformatic analysis of the correlation between *USP8* expression and immunogenic subtype (G1: immune “hot: tumor; G2: immune “cold” tumor) using the TCGA database. **c** Overall survival (OS) of pancreatic tumor patients with high or low expression of USP8 (n = 101). Wilcox tests method is indicated in **a** and Kaplan–Meier method is indicated in **c**.

**Supplementary Fig.2** USP8 inhibition significantly reduces the migration and invasion of KPC and BxPC-3 cells. **a** Effects of USP8 inhibition on the migration of KPC and BxPC-3 cells and quantification (**b**) of migration. Scale bar = 250μm. **c**, Effects of USP8 inhibition on the invasion of KPC and BxPC-3 cells and quantification (**d**) of invasion. Scale bar = 250μm. **e**, **f** WB analysis of EMT-related proteins in KPC and BxPC-3 cells after treatment with the USP8 inhibitor (1 μM, 24 h). The results are shown as the means ± SD of a representative experiment in **b** and **d**. The data are representative of three independent experiments. **p* < 0.05, ***p* < 0.01, ****p* < 0.001 assessed via a two-tailed t-test; ns: not significant.

**Supplementary Fig.3** The connection of *USP8* expression with immunosuppressive factors. **a** Bioinformatic analysis of the correlation between immunosuppressive factors and *USP8* across all tumor samples from the TCGA database. **b**, **c** Bioinformatic analysis of the correlation between immunosuppressive factors and *USP8* in pancreatic cancer using the TCGA database.

**Supplementary Fig.4** USP8 deficiency significantly improves anti-tumor immunogenicity. **a** IHC staining and further quantification (**b**) of Ki67 staining, and Casepase3 staining(n=7). Scale bars= 250μm. **c-f** Results of activated T cell-mediated tumor cell killing. Representative images and further quantification of living cells. The results are shown as means ± SD from one representative experiment in **b**, **d**, and **f**. The data are representative of three independent experiments. **p* < 0.05, ***p* < 0.01, ****p* < 0.001, *****p* < 0.0001 assessed via a two-tailed t-test; ns: not significant.

**Supplementary Fig.5** The USP8 inhibitor exerts a similar tumor suppressive effect in different molecular subtypes of pancreatic cancer. **a** Western blot results validating *Kras^G12D^* KD in the KPC cell line. **b** Schematic of the protocol of USP8 inhibitor (100 μg per mouse) for mice implanted subcutaneously with KPC parental cells and *Kras^G12D^* KD cells (5 ×10^5^). **c** Photographs of tumors removed from mice of each group (n = 5). **d** Curves showing the tumor growth in mice treated with the USP8 inhibitor. **e** The statistical plot of tumor weights of the four groups (n = 5). The results are shown as means ± SD from one representative experiment in **d**, **e**. The data are representative of three independent experiments. **p* < 0.05, ***p* < 0.01, ****p* < 0.001 assessed via a two-tailed t-test; ns: not significant.

**Supplementary Fig.6** USP8 interacts positively with PD-L1 in BxPC-3 cells and human pancreatic tumor tissues. **a** Photographs of immunofluorescence staining showing the interaction of USP8 and PD-L1 in BxPC-3 cells and fluorescence intensity plots (**b**) showing the co-localization of USP8 and PD-L1. The images are representative of three independent experiments. **c** Photographs of immunofluorescence staining showing the interaction of USP8 and PD-L1 in human pancreatic tumor tissues and fluorescence intensity plots (**d**) showing the co-localization of USP8 and PD-L1. The images are representative of three independent experiments.

**Supplementary Fig.7** USP8 inhibits PD-L1 ubiquitination-regulated proteasomal degradation. **a** USP8 activity in BxPC-3 cells by the HA-Ub-VS experiments treated with the USP8 inhibitor (DUB-IN-2) (1 μM, 4h). **b**, **c** PD-L1 levels in pancreatic cancer SW1990 cells treated with a concentration gradient and time gradient of the USP8 inhibitor, as assessed using western blotting. The image is representative of three independent experiments. **d**, **e** Western blotting and Flow cytometry of PD-L1 expression in pancreatic cancer BxPC-3 cells after treatment with *USP8* overexpression. A representative image of three independent experiments is shown. **f** The level of PD-L1 after treatment with the USP8 inhibitor (1 μM, 24 h) in BxPC-3 cells treated with MG132 (5 μM, 12 h), as assessed using western blotting. A representative image of three independent experiments is shown. **g** The level of PD-L1 expression in MG132 (5 μM, 12 h)-treated parental and *USP8* KD BxPC-3 cells, as assessed using western blotting. A representative image of three independent experiments is shown. **h** Analysis of PD-L1 stability in cycloheximide (CHX) (200 μg/mL) pretreated BxPC-3 cells incubated with the USP8 inhibitor (1 μM, 24 h). A representative image of three independent experiments is shown. **i** Analysis of PD-L1 stability in cycloheximide (CHX) (200 μg/mL) treated parental and *USP8* KD KPC cells. A representative image of three independent experiments is shown. **j**, **k** A densitometer was used to quantify the intensity of PD-L1 protein expression and the results are representative of three independent experiments. **l** Ubiquitination assay of PD-L1 in BxPC-3 cells. The cells were treated with MG132 (5 μM, 12 h) followed by USP8 inhibitor (1 μM, 24 h) treatment and then western blotting was used to detect PD-L1 and ubiquitin. **m** Ubiquitination assay of PD-L1 in parental and *USP8* KD BxPC-3 cells. Cells were treated with MG132 (5 μM, 12 h) then western blotting was used to detect PD-L1 and ubiquitin. Results are shown as the means ± SD of a representative experiment in **e**, **j**, and **k**. The data are representative of three independent experiments. **p* < 0.05, ***p* < 0.01, ****p* < 0.001 assessed via a two-tailed t-test; ns: not significant.

**Supplementary Fig.8** USP8 inhibition and depletion do not affect EGFR expression in PDAC. **a**-**d** USP8 inhibition (1 μM, 24 h) or depletion did not affect EGFR expression in KPC and BxPC-3 cells. **e** USP8 inhibition (100 μg per mouse, 3 times a week) did not affect EGFR expression in KPC pancreatic tumors.

**Supplementary Fig.9** USP8 inhibition via regulation of PD-L1 promotes pancreatic tumor immunotherapy in a subcutaneous tumor model. **a** Changes in the bodyweight of mice and the relative mouse body weight (**b**) on the last day (n = 5). **c** Representative images displaying spleens removed from subcutaneous KPC cells bearing mice treated with USP8 inhibitor, αPD-L1, or their combination (n = 3). **d** The statistical plot of spleen weights of the four groups(n=3). **e** Blood biochemical tests to evaluate the safety of the drugs. **f**, **g** Representative images of IHC staining and quantification of PD-L1 expression, Ki67 staining, and Casepase3 staining(n=5). Scale bars=250μm. **h**, **i** Results of activated T cell-mediated tumor cell killing. Representative images and further quantification of living cells. The results are shown as means ± SD from one representative experiment in **a**, **b**, **d**, **e**, **g**, and **i**. The data are representative of three independent experiments. **p* < 0.05, ***p* < 0.01, ****p* < 0.00, *****p* < 0.0001 assessed via a two-tailed t-test; ns: not significant.

**Supplementary Fig.10** USP8 deficiency combined with αPD-L1 promotes pancreatic tumor immunotherapy. **a**, **b** Statistical analysis of MHC-1 levels in pancreatic cancer KPC cells treated with *Usp8* KD and subjected to flow cytometry. A representative image of three independent experiments is shown. **c**, **d** Representative images of IHC staining and quantification of Ki67 staining and Casepase3 staining(n=5). Scale bars=250μm. The results are shown as means ± SD from one representative experiment in **b** and **d**. The data are representative of three independent experiments. **p* < 0.05, ***p* < 0.01, ****p* < 0.00, *****p* < 0.0001 assessed via a two-tailed t-test; ns: not significant.

**Supplementary Fig.11** USP8 inhibition via regulation of PD-L1 promotes pancreatic tumor immunotherapy in an orthotopic tumor model. **a** Changes in the bodyweight of mice and the relative mouse body weight (**b**) on the last day (n = 5). **c**, **d** Statistical analysis of MHC-1 levels in pancreatic cancer KPC cells treated with the USP8 inhibitor (1 μM, 24 h) and subjected to flow cytometry. A representative image of three independent experiments is shown. **e** Western blotting of PD-L1 levels in an orthotopic tumor model, statistical results (**f**) shown. **g**, **h** Representative images of IHC staining and quantification of PD-L1 expression, Ki67 staining, and Casepase3 staining(n=5). Scale bars= 250μm. The results are shown as means ± SD from one representative experiment in **a**, **b**, **d**, **f**, and **h**. The data are representative of three independent experiments. **p* < 0.05, ***p* < 0.01, ****p* < 0.00, *****p* < 0.0001 assessed via a two-tailed t-test; ns: not significant.

**Supplementary Fig.12** Inhibitory effect of the pancreatic tumor growth using a USP8 inhibitor combined with αPD-L1 or αCTLA-4. **a** Schematic of the protocol for USP8 inhibitor and αPD-L1 or αCTLA-4 combination therapy for orthotopic KPC cell (5 ×105)-bearing mice. **b** Photographs of tumors removed from mice of each group (n = 5). **c** The statistical plot of tumor weights of the four groups (n = 5). **d** Flow cytometry of PD-L1 levels in an orthotopic tumor model, statistical results (**e**) shown. **f** Flow cytometry of MHC-1 levels in an orthotopic tumor model, statistical results (**g**) shown.

**h**, **i** Flow cytometry of CD3+ T cells, CD8+ T cells, CTLA-4+CD8+ T cells, IFN-γ+CD8+ T cells, GranzymeB+CD8+ T cells in the tumor region and the statistical analysis of the results (n = 5). The results are shown as means ± SD from one representative experiment in **c**, **e**, **g**, and **i**. The data are representative of three independent experiments. **p* < 0.05, ***p* < 0.01, ****p* < 0.00, *****p* < 0.0001 assessed via a two-tailed t-test; ns: not significant.

**Supplementary Fig.13** The distribution of metastases to the liver, lung, spleen, and lymph nodes. **a**, **b** Representative images showing the lung and liver. **c** Representative images showing spleens with tumor metastasis and a statistical table (**d**) of the percentage of spleens with tumorigenesis. **e** Representative images showing abdominal lymph nodes with tumor metastasis and a statistical table (**f**) of the percentage of spleens with tumorigenesis.

**Supplementary Fig.14** Analysis of activated T cell-mediated tumor cell killing by the combination therapy in *Cd274* KO KPC cells compared to parental KPC cells *in vitro*. **a, b** Results of activated T cell-mediated tumor cell killing. Representative images and further quantification of living cells. The results are shown as means ± SD from one representative experiment in **b**. The data are representative of three independent experiments. **p* < 0.05, ***p* < 0.01, ****p* < 0.00 assessed via a two-tailed t-test; ns: not significant.

**Supplementary Fig.15 a, b** Gating strategy to analyze the level of PD-L1 and MHC-1 in pancreatic cancer cells. **c** Gating strategy to analyze the level of PD-L1 and MHC-1 in orthotopic KPC cell-bearing mice. **d** Gating strategy to analyze the number and function of tumor-infiltrating lymphocytes (TILs) in orthotopic KPC cell-bearing mice.
