# Supplementary material for: Targeting ubiquitin-specific protease 8 sensitizes anti-programmed death-ligand 1 immunotherapy of pancreatic cancer
Source: Cell Death Differ. 2022 Dec 20;30(2):560–75. doi: 10.1038/s41418-022-01102-z (PMC9950432; doi:10.1038/s41418-022-01102-z)
Supplement: Supplementary file 19 — Original Data File [file 41418_2022_1102_MOESM19_ESM.pdf]

Fig. 1g

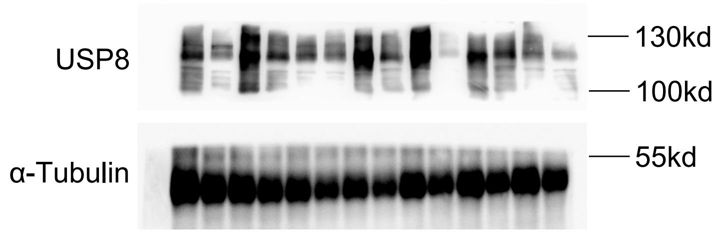

Fig. 1i

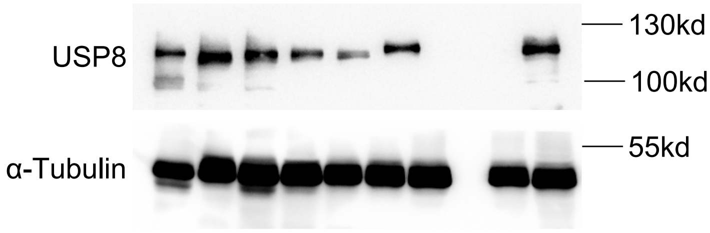

Fig. 1

Fig. 3e

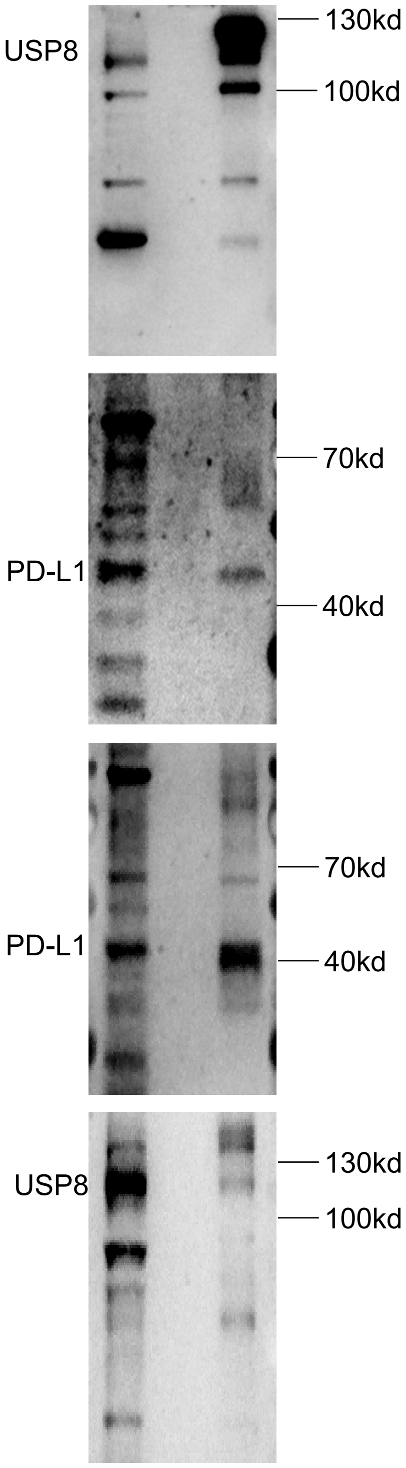

Fig. 3f

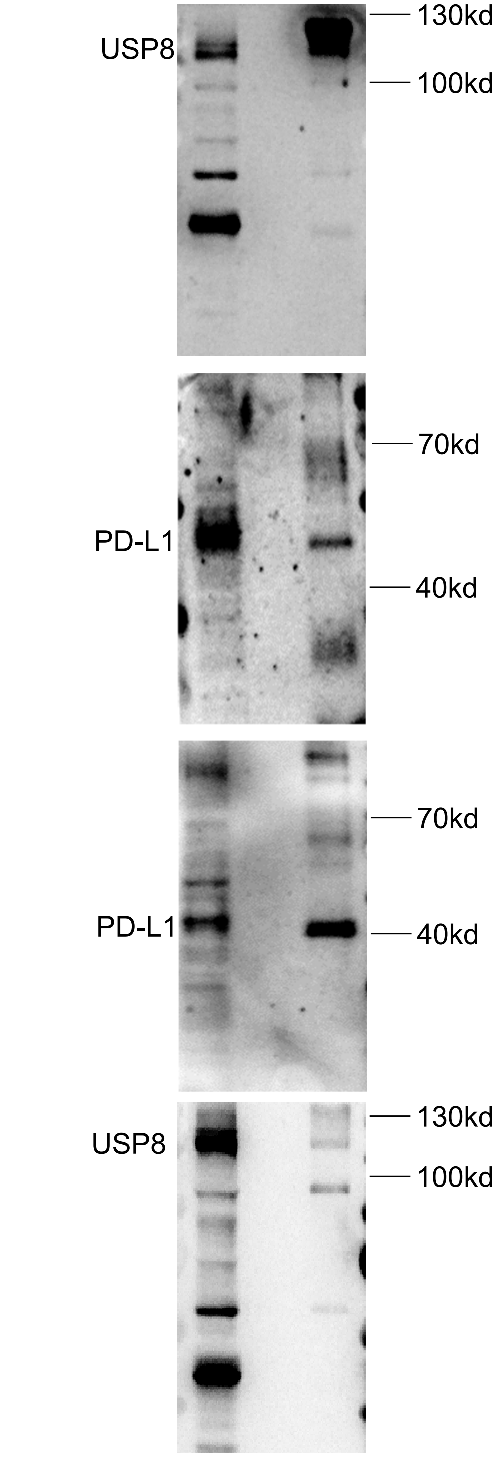

Fig. 3g

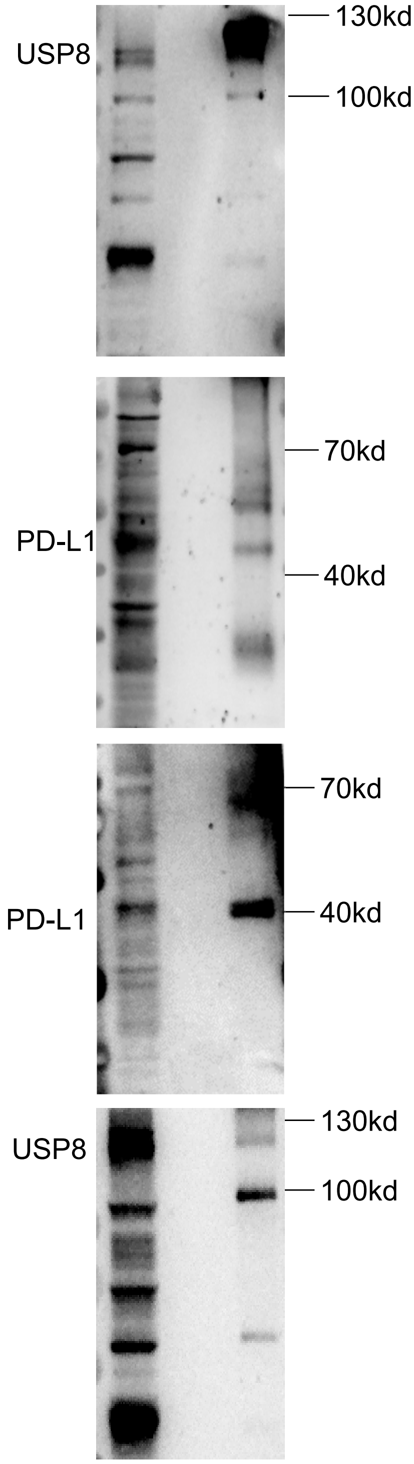

Fig. 3h

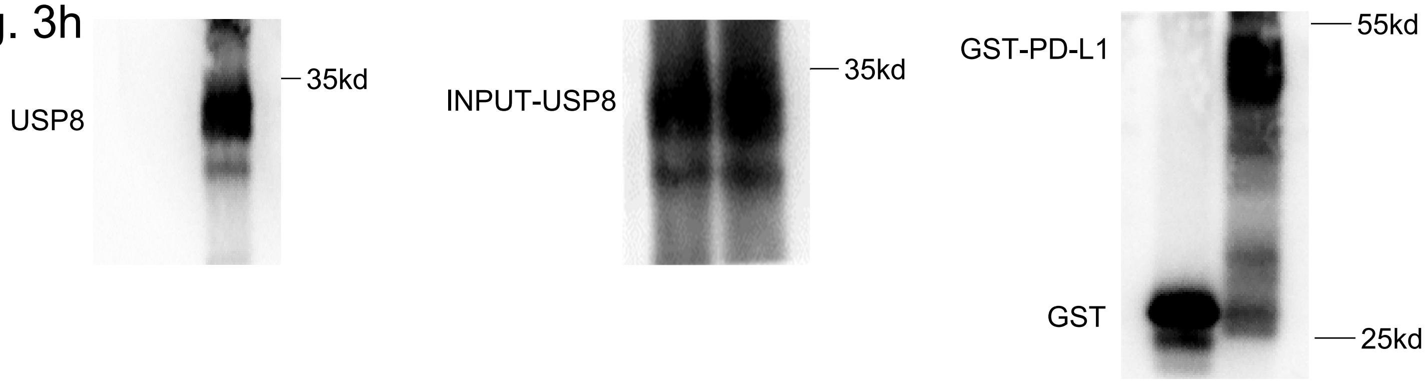

Fig. 3

Fig. 4a

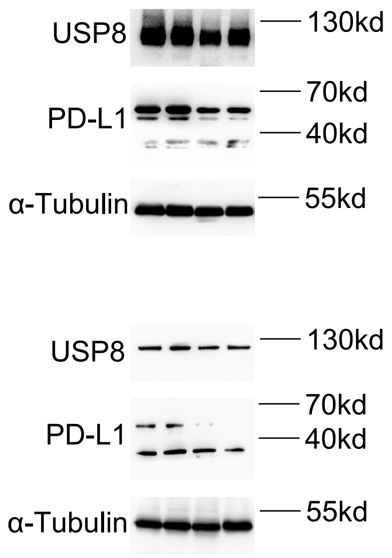

Fig. 4b

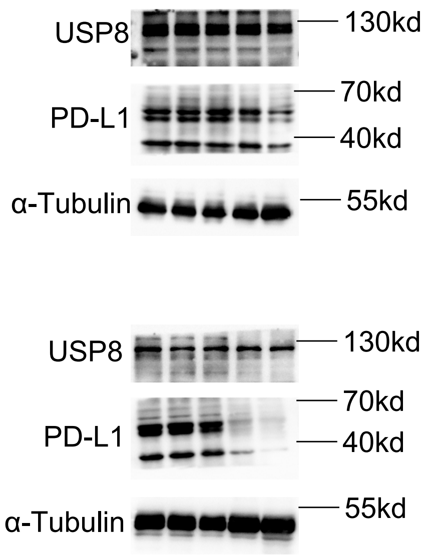

Fig. 4c

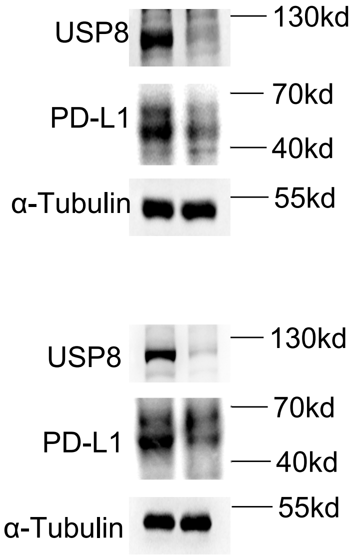

Fig. 4h

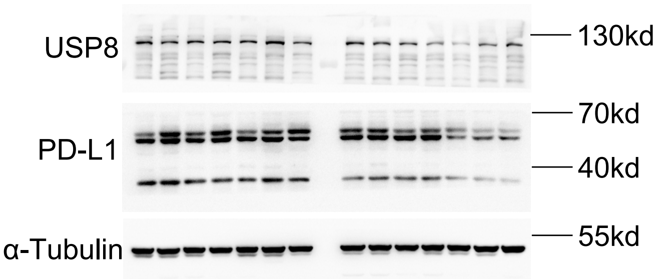

Fig. 4m

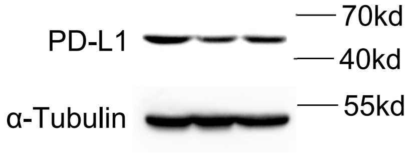

Fig. 4n

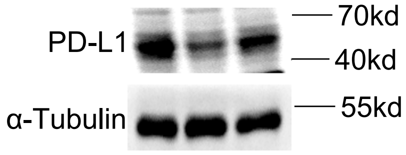

Fig. 4o

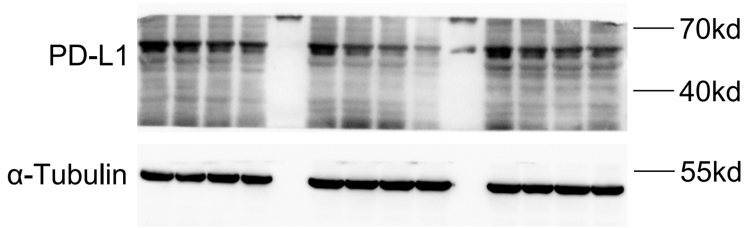

Fig. 4p

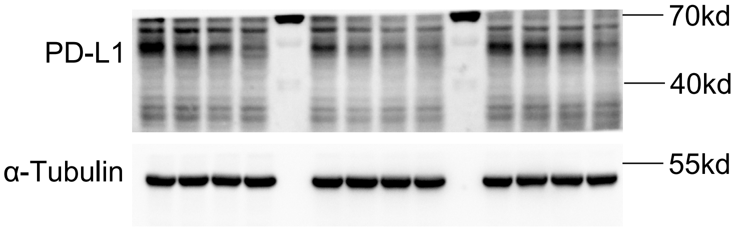

Fig. 4s

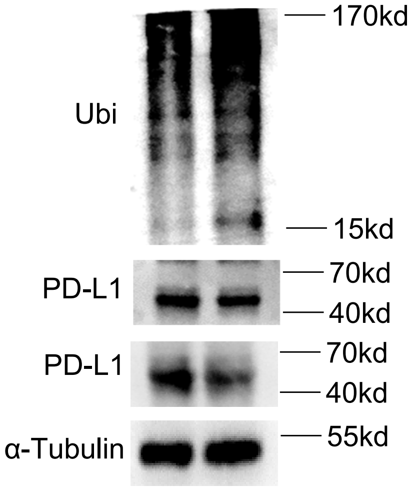

Fig. 4t

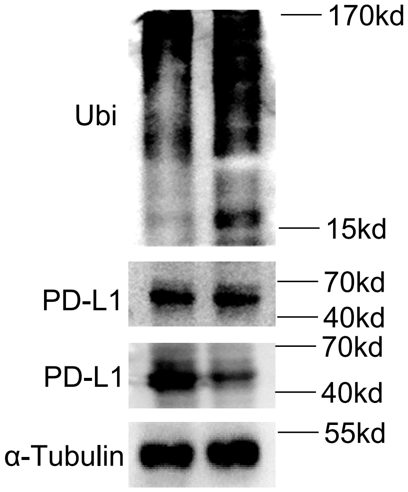

Fig. 4

Fig. 5g

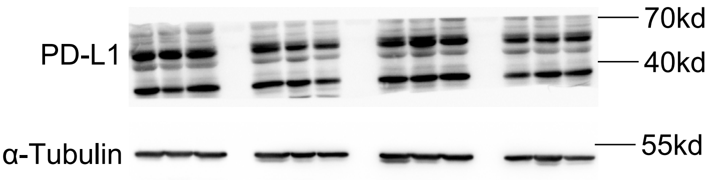

Fig. 8a

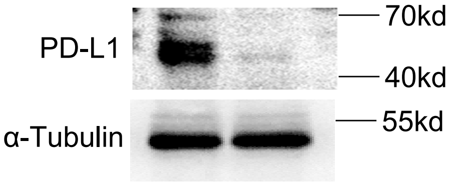

Fig. 8

Fig. S2e

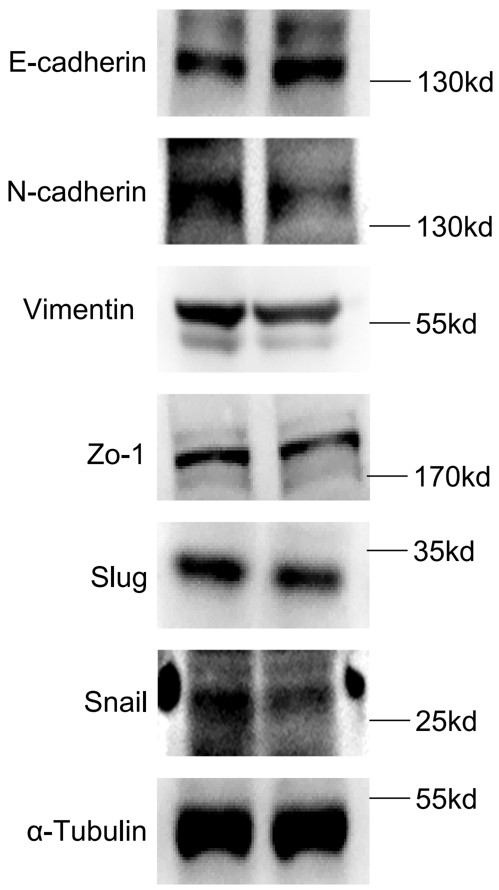

Fig. S2f

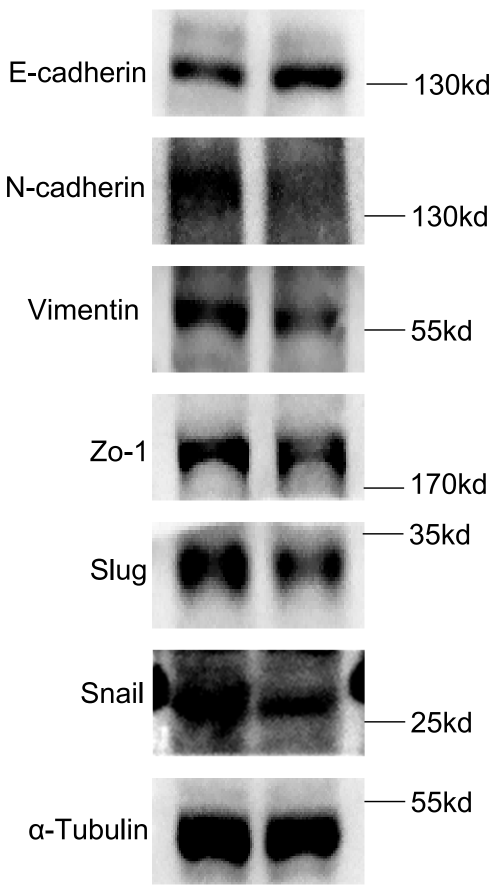

Supplementary Fig. 2

Fig. S5a

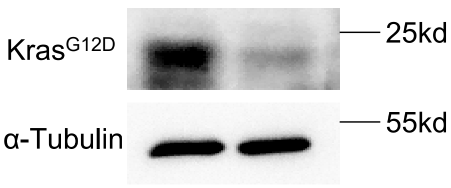

Supplementary Fig. 5

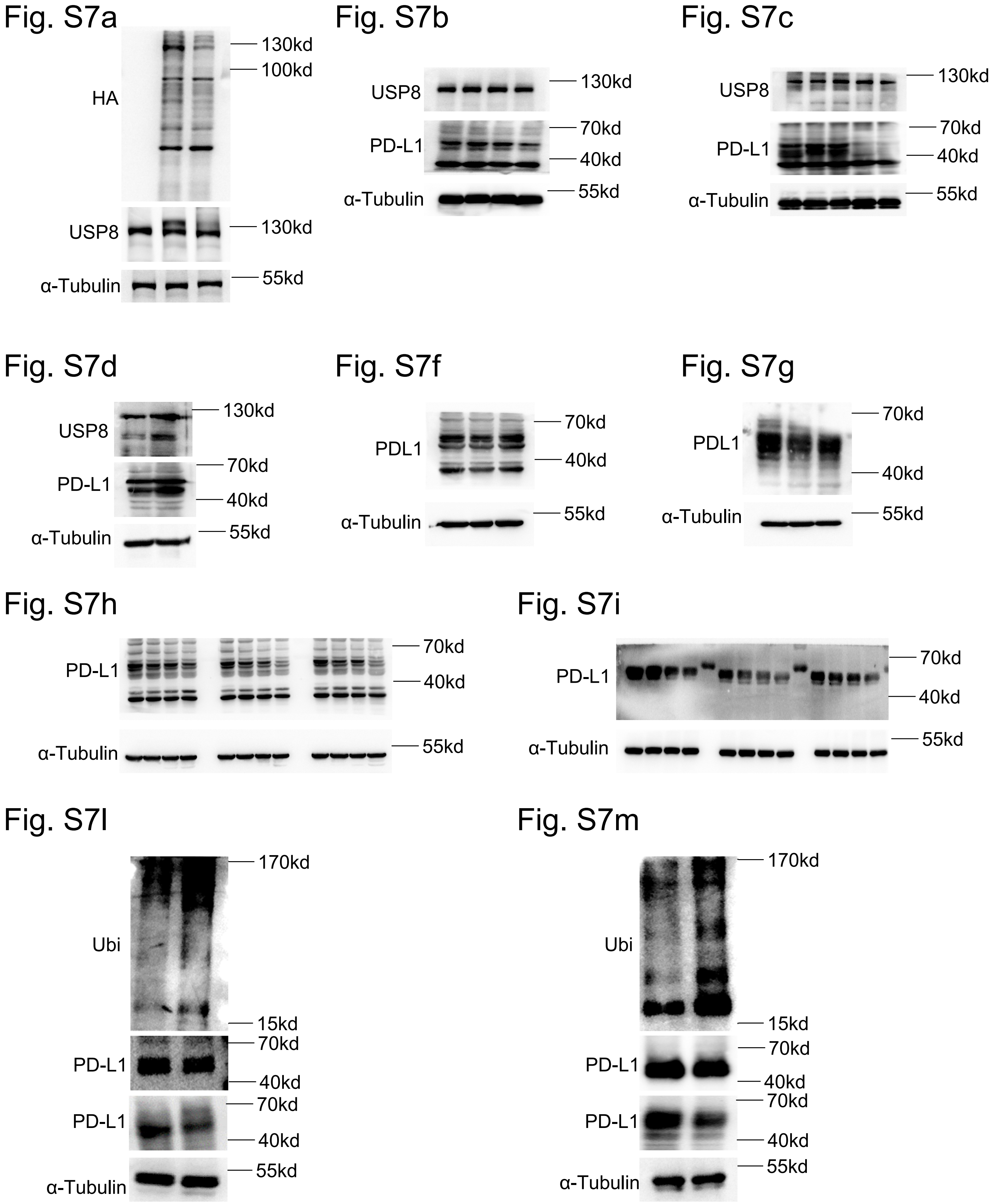

Supplementary Fig. 7

Fig. S8a

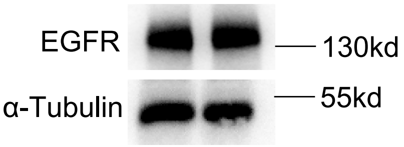

Fig. S8b

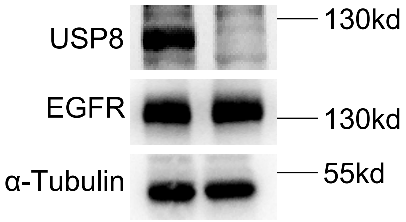

Fig. S8c

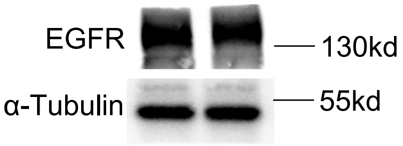

Fig. S8d

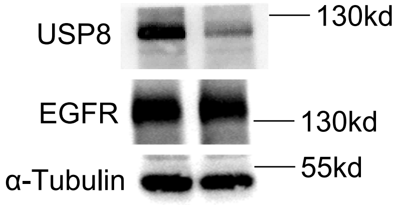

Fig. S8e

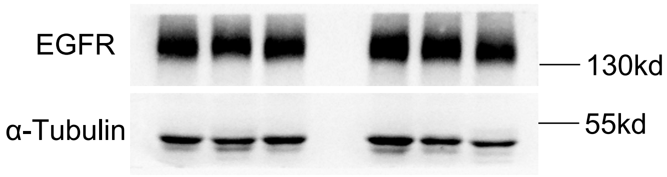

Supplementary Fig. 8

Fig. S11e

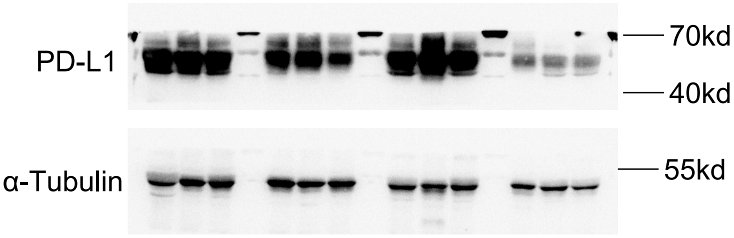

Supplementary Fig. 11
